# A Role for CF1A 3′ End Processing Complex in Promoter-Associated Transcription

**DOI:** 10.1371/journal.pgen.1003722

**Published:** 2013-08-15

**Authors:** Nadra Al Husini, Paul Kudla, Athar Ansari

**Affiliations:** Department of Biological Sciences, Wayne State University, Detroit, Michigan, United States of America; Stanford University School of Medicine, United States of America

## Abstract

The Cleavage Factor 1A (CF1A) complex, which is required for the termination of transcription in budding yeast, occupies the 3′ end of transcriptionally active genes. We recently demonstrated that CF1A subunits also crosslink to the 5′ end of genes during transcription. The presence of CF1A complex at the promoter suggested its possible involvement in the initiation/reinitiation of transcription. To check this possibility, we performed transcription run-on assay, RNAP II-density ChIP and strand-specific RT-PCR analysis in a mutant of CF1A subunit Clp1. As expected, RNAP II read through the termination signal in the temperature-sensitive mutant of *clp1* at elevated temperature. The transcription readthrough phenotype was accompanied by a decrease in the density of RNAP II in the vicinity of the promoter region. With the exception of TFIIB and TFIIF, the recruitment of the general transcription factors onto the promoter, however, remained unaffected in the *clp1* mutant. These results suggest that the CF1A complex affects the recruitment of RNAP II onto the promoter for reinitiation of transcription. Simultaneously, an increase in synthesis of promoter-initiated divergent antisense transcript was observed in the *clp1* mutant, thereby implicating CF1A complex in providing directionality to the promoter-bound polymerase. Chromosome Conformation Capture (3C) analysis revealed a physical interaction of the promoter and terminator regions of a gene in the presence of a functional CF1A complex. Gene looping was completely abolished in the *clp1* mutant. On the basis of these results, we propose that the CF1A-dependent recruitment of RNAP II onto the promoter for reinitiation and the regulation of directionality of promoter-associated transcription are accomplished through gene looping.

## Author Summary

The termination of transcription requires two major multisubunit complexes in budding yeast. These termination complexes are localized at the 3′ end of genes. Recent studies have found the termination factors occupying the 5′ end of genes as well. In this study, we investigate the physiological role of a termination factor at the 5′ end of a gene. Our results show that the CF1 termination complex affects the recruitment of the transcription enzyme RNAP II onto the promoter for reinitiation of transcription. The complex also affects the directionality of transcription of the promoter-bound polymerase. We also found that the looped gene conformation was disrupted in the absence of a functional termination complex. The overall conclusion of these results is that the terminator-bound factors contact the 5′ end of genes due to gene looping, and affect both the recruitment of the polymerase at the promoter for reinitiation, and directionality of the promoter-initiated transcription. Thus, the role of termination factors is not restricted to the 3′ end of the gene, but they are also involved in promoter-associated transcription.

## Introduction

The process of transcription can be divided into three principal steps; initiation, elongation and termination [1]. The accomplishment of each of these steps during the RNAP II-mediated transcription cycle requires a number of accessory factors. The initiation of transcription requires gene specific transcription factors as well as general transcription factors (GTFs): TFIID, TFIIB, TFIIA, TFIIF, TFIIE, TFIIH and Mediator complex, that assemble on the promoter to form the preinitiation complex [2]–[5]. The termination of transcription, which is intimately linked to the cleavage and polyadenylation of precursor mRNA, exhibits a similar requirement for a group of termination factors organized into two macromolecular complexes called Cleavage-Polyadenylation-Factor (CPF) complex and Cleavage Factor-1 (CF1) complex in yeast [6]–[10]. The initiation and termination factors have been remarkably conserved during evolution. The generally accepted view is that the initiation factors operate exclusively at the 5′ end of a gene and are committed to starting the transcription cycle, while termination factors have a dedicated role in ending the transcription cycle at the 3′ end of a gene. A number of recently published reports, however, challenge this dogma. It is evident that at least some initiation factors are also necessary for termination, and the termination factors likewise may have a role in the initiation or reinitiation step of the transcription cycle [10]–[14].

An increasing amount of biochemical, genetic and functional evidence suggest the existence of a network of complex interactions between initiation and termination factors. The general transcription factor TFIIB, for example, exhibits multiple genetic and physical interactions with the factors operating at the 3′ end of genes [15]–[18]. These studies suggested a plausible role for TFIIB in the termination process. Accordingly, it was recently demonstrated that TFIIB is indeed actively engaged in termination of transcription in mammals and flies [17], [19]. Yeast Mediator subunit Srb5, which has a well-established function in the initiation of transcription, likewise, crosslinks to the 3′ end of genes and participates in the termination process [20]. TFIID is another promoter-bound protein that contacts the factors operating at the 3′ end of genes. Biochemical analysis of mammalian TFIID has revealed its reciprocal interaction with the CPSF 3′ end processing complex [21]. The TFIID-CPSF interaction is evolutionarily conserved. A recent proteomic analysis of yeast TFIID complex identified multiple interactions of TFIID subunit TAF150 with the components of the CPF 3′ end processing complex, which is the yeast homologue of CPSF complex [22]–[24].

Like initiation factors, an array of termination factors also crosstalk with the 5′ end of genes. The foremost among them is Ssu72, which was discovered as a protein of unknown function that genetically interacts with TFIIB [15]. Later on, yeast proteomic analysis identified Ssu72 as a component of the CPF 3′ end processing complex [25]–[27]. Ssu72 crosslinks to the 5′ end of genes, and interacts with several promoter-bound factors [22], . Pta1, which is a subunit of CPF complex, and Rat1 are other terminator-bound factors that physically interact with the 5′ end of genes and the associated initiation factors [30], [35]. Besides CPF complex, CF1 complex is also required for both the cleavage-polyadenylation of mRNA as well as termination of transcription. At least three subunits of this complex (Rna14, Rna15 and Pcf11) associate with both ends of a transcriptionally engaged gene [18], [36]. CF1A subunits exhibit genetic and physical interaction with several promoter-bound factors that include both the general transcription factors and gene specific factors [16], [18], [33], [37]–[41]. Furthermore, CF1A subunits are also required for juxtaposition of the promoter and terminator regions to form a looped gene structure [18]. The well-orchestrated interaction of the distal ends of a gene strongly suggests that the termination and initiation steps of transcription may operate in a cooperative manner.

The presence of termination factors on the promoter region could influence the events taking place at the 5′ end of genes. One possible role of the termination factors at the 5′ end could be to regulate initiation or reinitiation of transcription. It was recently demonstrated that proper termination of transcription is required for efficient execution of the transcription cycle in mammalian cells [42]. In that study, a termination defect adversely affected the recruitment of the general transcription factors onto the promoter of the same gene leading to a decrease in initiation of transcription. In a related study, a decrease in the density of RNAP II at the promoter region was observed in the termination-defective Ssu72-C15S mutant [43]. One possible interpretation of these results is that proper termination is important for the recruitment of polymerase at the promoter for reinitiation. It is conceivable that the physical proximity of the promoter and terminator regions, which results in a looped gene conformation, facilitates a direct transfer of the released polymerase from the 3′ end to the juxtaposed promoter [30]. This would help bypass the rate-limiting step of recruitment of polymerase on the promoter, leading to enhanced transcription of the gene. A transfer of polymerase molecules from the terminator to the promoter has, indeed, been shown for RNAP III-transcribed genes [44]. We propose that a similar termination-reinitiation coupling is taking place during RNAP II-mediated transcription as well. Another possible function of termination factors at the 5′ end of genes could be in providing directionality to the promoter-bound RNAP II to transcribe the sense strand. Genome wide analysis of human and yeast systems revealed the unexpected finding that RNAP II tends to transcribe both in the sense as well as anti-sense direction from the promoter region [45]–[48]. The promoter initiated anti-sense transcription, however, is aborted, thereby favoring productive elongation of the sense transcript. What confers directionality to the promoter-bound polymerase remains unclear. A recent study carried out in budding yeast demonstrated that the termination factors inhibit transcription of the promoter-initiated anti-sense transcripts, thereby providing directionality to the promoter-bound polymerase [49].

Here we demonstrate the role of CF1A complex in the promoter-associated transcription in budding yeast. In a mutant of Clp1 subunit of the CF1A complex, recruitment of the whole CF1A complex at the 3′ end of genes was compromised, leading to a termination defect. The termination defect coincided with a decrease in the recruitment of RNAP II on the promoter indicating a possible initiation defect. Since there was no significant decrease in the recruitment of the general transcription factors onto the 5′ end of a gene in the *clp1* mutant, these results strongly suggest a novel role for the CF1A complex in reinitiation of transcription. We further found a role for CF1A complex in the inhibition of promoter-initiated anti-sense transcription. Thus, CF1A complex may have an additional function in providing directionality to bivalent yeast promoters. The CF1A-dependent promoter-based events coincide with the gene assuming a looped conformation, thereby suggesting a possible role of gene looping in reinitiation of transcription in the sense direction.

## Results

CF1A is a hexameric complex comprised of two subunits each of Rna14 and Rna15, and one subunit each of Pcf11 and Clp1 [50]. The Rna14, Rna15 and Pcf11 subunits have been studied extensively due to the availability of conditional mutant alleles. In contrast, little is known about the physiological role of Clp1. Recent studies, however, have implicated Clp1 both in the 3′ end processing of precursor mRNA and in the termination of transcription [51]–[53]. Structural analysis using mutants revealed that Clp1 makes a direct physical contact with the Pcf11 subunit of CF1A complex as well as with the Ssu72 and Ysh1 subunits of CPF complex [50]–[52].

### Clp1 is required for the recruitment of a termination-competent CF1A complex on transcriptionally active genes

**Figure 1:**
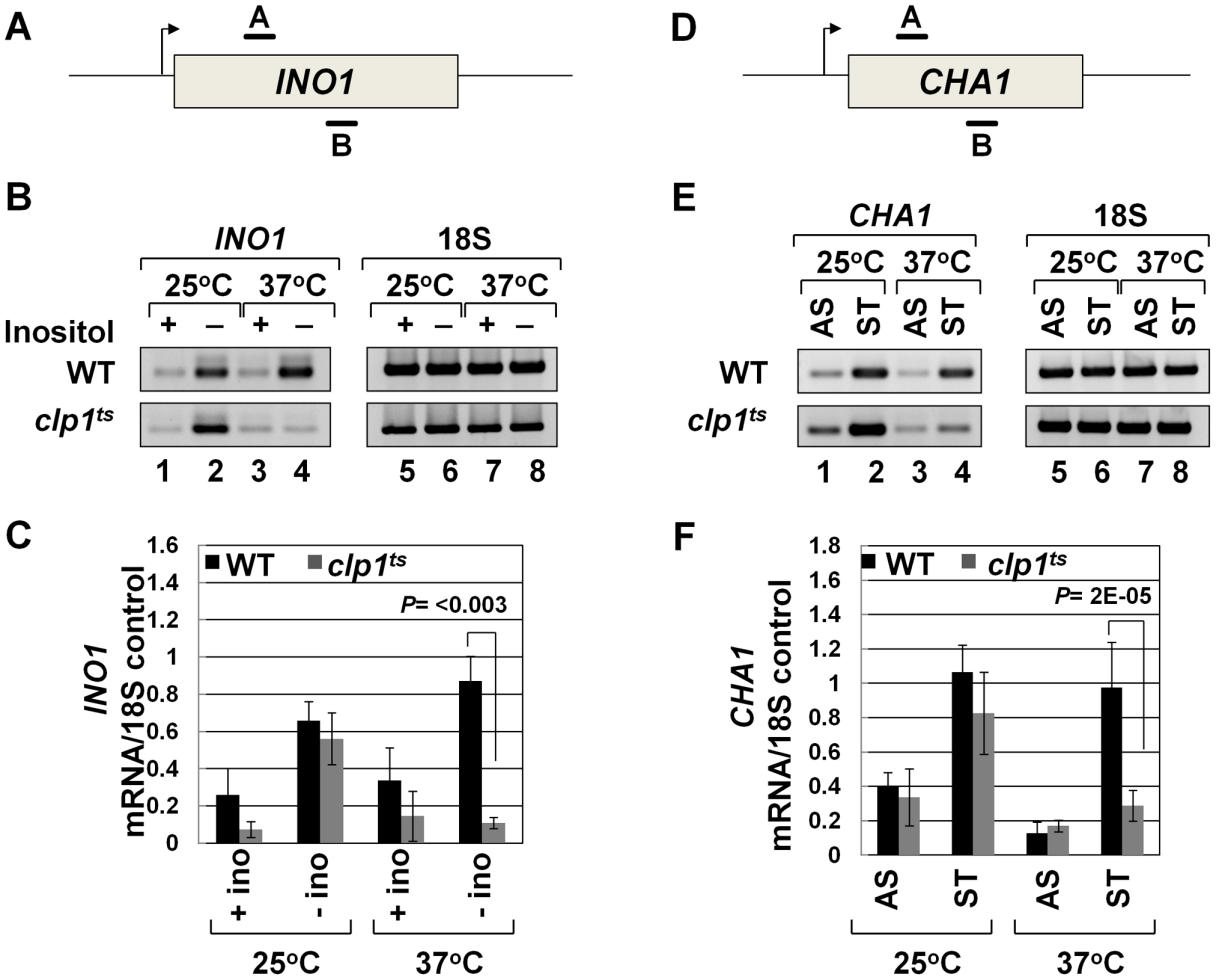
Transcript levels of *INO1* and *CHA1* are adversely affected in the *clp1^ts^* mutant. (A, D) Schematic representations of *INO1* and *CHA1* showing the positions of the primer pairs used in RT-PCR analysis. (B) and (E) RT-PCR analysis of *INO1* and *CHA1* in *clp1^ts^* and isogenic wild type strains during induced and non-induced transcription at 25°C and 37°C. (C) and (F) Quantification of the data shown in B and E respectively. The transcript level of 18S was used as the control. The results shown are an average of four PCRs from two cDNA preparations from two different RNA extractions. Error bars indicate one unit of standard deviation. AS = Ammonium sulfate, ST = Serine/Threonine, WT = Wild type.

To further analyze the role of Clp1 in transcription, we used a temperature-sensitive mutant of the factor called *clp1-769-5* [54]. Western blot analysis revealed that the Clp1 protein almost completely disappeared from the mutant cells following the temperature shift to 37°C, but there was only a marginal change in the signal for other CF1A subunits at the elevated temperature (Figure S1). We examined the transcription of *INO1* and *CHA1* in the mutant *clp1* strain in cells grown at the permissive (25°C) and non-permissive (37°C) temperatures. We chose *INO1* and *CHA1* for our study because their regulation is relatively well understood and their transcriptional state can be regulated by simply changing the growth conditions. Furthermore, *CHA1* is relatively isolated in the yeast genome and therefore is a good candidate to study upstream and downstream transcription by transcription run-on (TRO) assay. RT-PCR was carried out using primers A and B as shown in Figure 1A and 1D in the mutant and wild type strains at 25°C and 37°C. RT-PCR analysis revealed that the transcript level of both *INO1* and *CHA1* decreased by about 4–8 fold upon shifting the mutant cells to 37°C (Figure 1B and 1E, lane 4; Figure 1C and 1F). No such decrease in transcript level was observed upon shifting the wild type cells to elevated temperature (Figure 1B and 1E, lane 4; Figure 1C and 1F). Thus, Clp1 is essential for optimal transcription of both *INO1* and *CHA1* in yeast. Since there was no appreciable decrease in the amount of CF1A subunits Rna14, Rna15 and Pcf11 in the mutant cells at the elevated temperature (Figure S1), we next checked if CF1A complex is recruited at the 3′ end of genes in the mutant cells. ChIP analysis revealed that the recruitment of Rna14, Pcf11 and Rna15 at the 3′ end of *INO1* and *CHA1* exhibited a decline following the temperature shift to 37°C (Figure S2, B and D, lanes 4, 12 and 20). No such decrease in the recruitment of CF1A subunits was observed in the wild type cells at elevated temperature (Figure S3, B and D, lanes 4, 12 and 20). The overall conclusion of these results is that the normal expression of *INO1* and *CHA1* is dependent on Clp1, and that the recruitment of a functional CF1A complex at the 3′ end of these two genes occurs in a Clp1-dependent manner.

**Figure 2:**
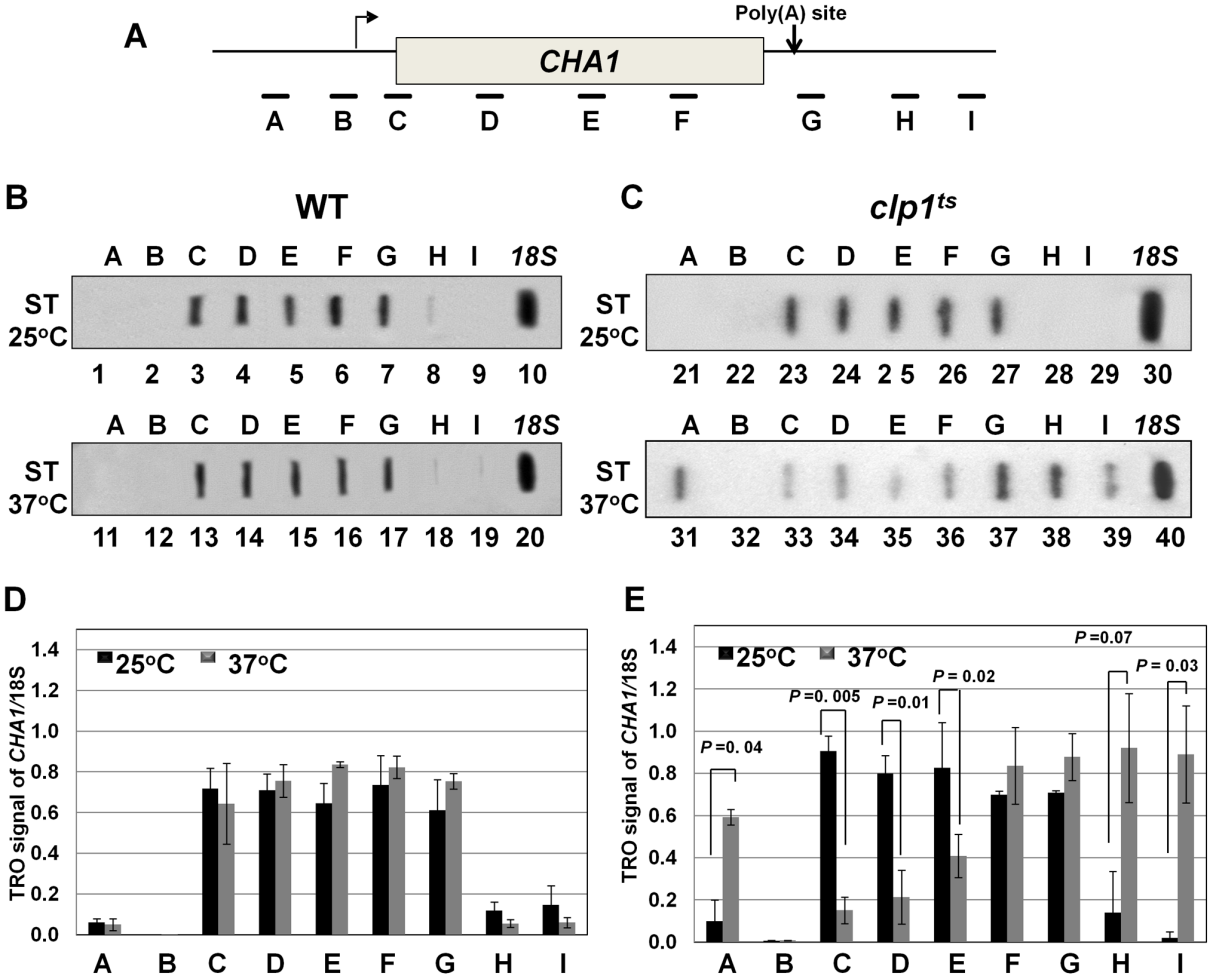
TRO analysis showing transcription readthrough and alteration in promoter-associated transcription in the *clp1^ts^* mutant. (A) Schematic depiction of the *CHA1* gene indicating the positions of the probes (A–I) used in the TRO assay. (B) TRO analysis of *CHA1* in the wild type strain at 25°C and 37°C under induced transcription of the gene. (C) TRO analysis of *CHA1* in the temperature-sensitive mutant of Clp1 at the permissive (25°C) and non-permissive (37°C) temperatures. (D) and (E) Quantification of the data shown in B and C respectively. Error bars indicate one unit of standard deviation. The results shown are an average of at least four independent replicates. ST = Serine/Threonine, WT = Wild type, TRO = Transcription run-on.

**Figure 3:**
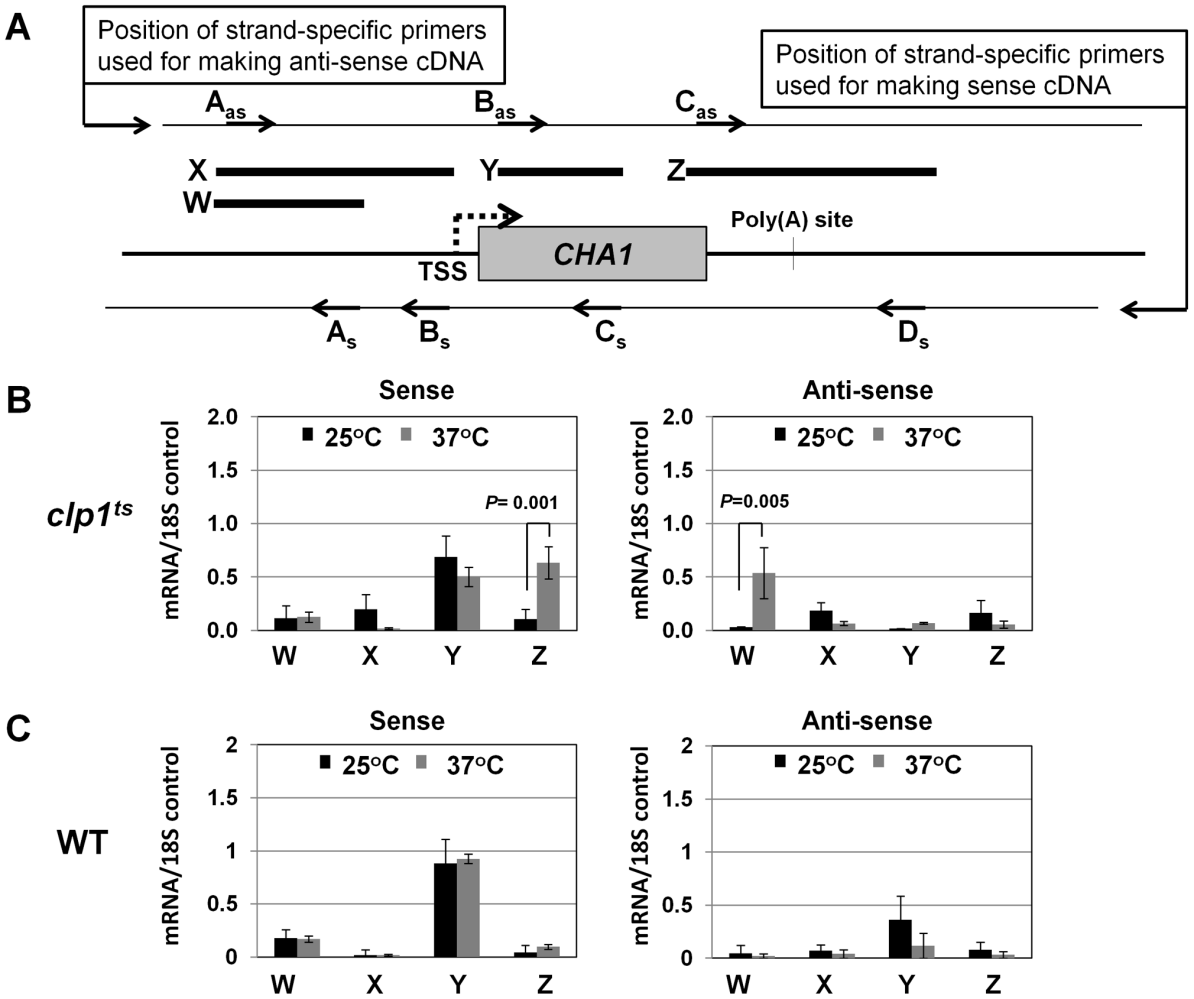
Strand specific RT-PCR showing transcription readthrough and promoter-initiated anti-sense transcription in the *clp1^ts^* mutant. (A) Schematic representation of the *CHA1* locus indicating the positions of primers used for reverse-transcribing sense transcripts (As, Bs, Cs and Ds) and anti-sense transcripts (Aas, Bas and Cas), and W, X, Y and Z regions amplified in RT-PCR analysis. (B) Strand-specific RT-PCR analysis of sense and anti-sense transcripts of *CHA1* in the *clp1* mutant at the permissive (25°C, black bars) and non-permissive (37°C, grey bars) temperatures. (C) Strand-specific RT-PCR analysis of sense and anti-sense transcripts of *CHA1* in the isogenic wild type strain at 25°C (black bars) and 37°C (grey bars). The results shown are an average of four PCRs from two cDNA preparations from two different RNA extractions. Error bars indicate one unit of standard deviation. TSS = Transcription start site, WT = Wild type, subscript s = sense and as = antisense.

To understand the role of Clp1 in the transcription cycle, we performed transcription run-on (TRO) analysis of *CHA1* in the wild type and temperature-sensitive *clp1-769-5* strains during different transcriptional states of the gene. The transcription of *CHA1* is regulated by the nitrogen source in the growth medium. The gene is maintained in a transcriptionally repressed state in a medium containing ammonium sulfate as the nitrogen source, and is stimulated upon shifting cells to a medium containing serine and threonine [55]. The position of transcriptionally active RNAP II was monitored at the positions A to I as shown in Figure 2A. The TRO analysis found transcriptionally active RNAP II being almost uniformly distributed between the promoter and the terminator regions of *CHA1* in the wild type strain during induced transcription (Figure 2B, lanes 3–7 and 13–17; Figure 2D). In the *clp1-769-5* mutant, however, the polymerase read through the termination signal into the downstream region at elevated temperature (Figure 2C, lanes 38 and 39; Figure 2E). No such transcription readthrough was observed in the mutant strain at the permissive temperature (Figure 2C, lanes 28 and 29; Figure 2E) or in the wild type cells at 37°C (Figure 2B, lanes 18 and 19; Figure 2D). Strand-specific RT-PCR analysis corroborated the presence of sense transcripts downstream of the termination signal of *CHA1* in the *clp1* mutant at elevated temperature (Figure 3B, region Z). No such readthrough transcripts were observed in the isogenic wild type strain under identical conditions (Figure 3C, region Z). Strand-specific RT-PCR analysis was carried out using primers shown in Figure 3A and described in the figure legend. These results confirmed the role of Clp1 in the termination of transcription in budding yeast.

### A functional CF1A complex is required for reinitiation of transcription

**Figure 4:**
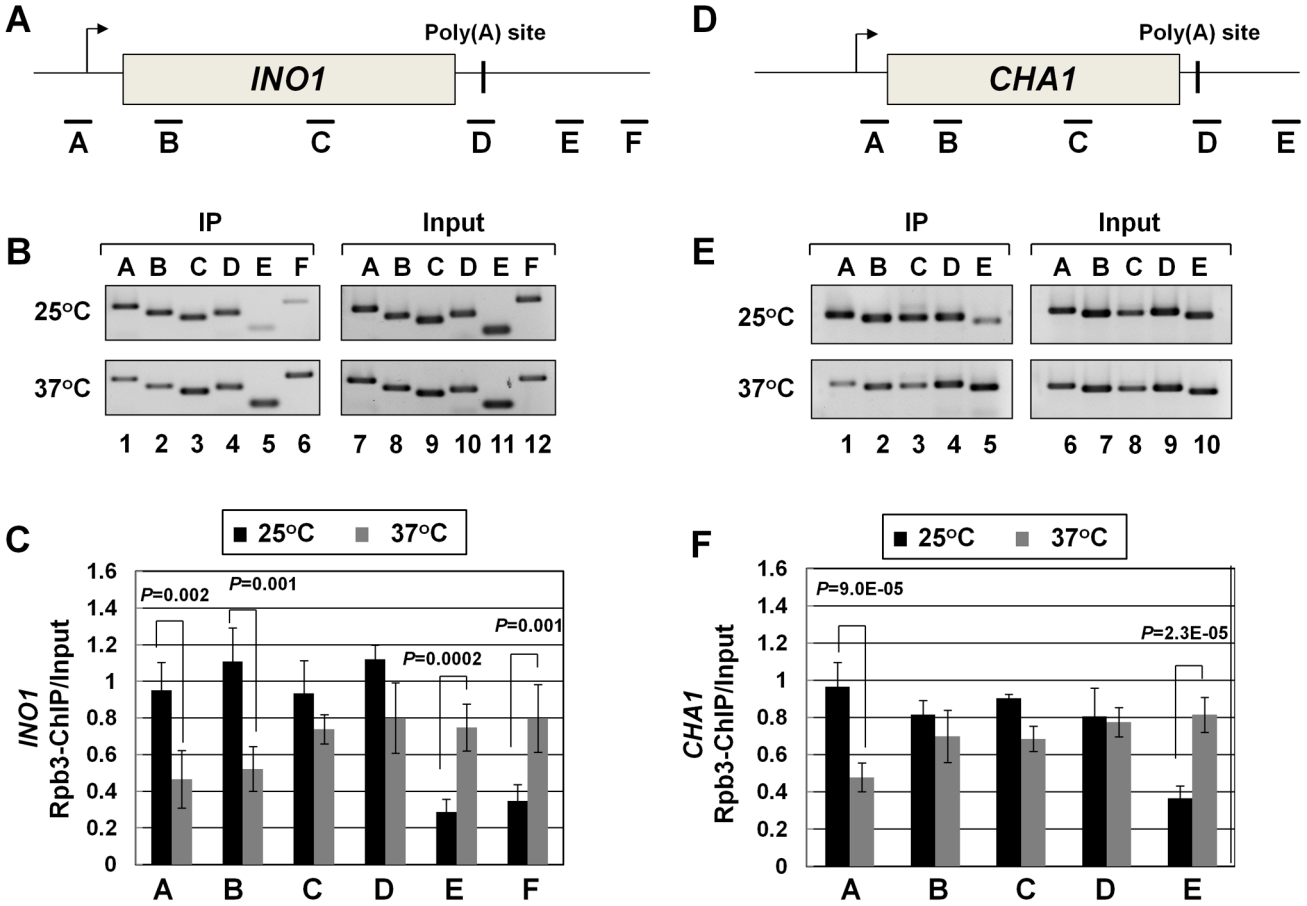
RNAP II density in the promoter region exhibits a decline in the *clp1^ts^* mutant. (A, D) Schematic depictions of *INO1* and *CHA1* showing the positions of ChIP primer pairs. (B, E) ChIP analysis showing polymerase density in different regions of *INO1* and *CHA1* in the *clp1* mutant at the permissive (25°C) and non-permissive (37°C) temperatures. (C and F) Quantification of data shown in B and E respectively. The input signals represent DNA prior to immunoprecipitation. The results shown are an average of at least eight independent PCRs from four separate immunoprecipitates from two independently grown cultures. Error bars indicate one unit of standard deviation. IP = immunoprecipitate.

Recently, we demonstrated crosslinking of Rna14, Rna15 and Pcf11 subunits of CF1A complex to the distal ends of genes in a transcription-dependent manner [18]. Here we show that the Clp1 subunit also localizes to both the 5′ and 3′ ends of transcriptionally active *INO1* and *CHA1* (Figure S4, B and E, lanes 1 and 4; Figure S4, C and F). The CF1A complex, being a cleavage-polyadenylation factor, is expected to bind to the 3′ end of genes. It was, however, intriguing to find the entire CF1A complex occupying the 5′ end of genes as well. A clue regarding the role of the CF1A complex at the 5′ end of genes came when we observed that the transcription readthrough phenotype of the mutant strain at the elevated temperature was accompanied by a decrease in the TRO signal in the promoter-proximal coding region (Figure 2C, lane 33). This result strongly suggested a role for Clp1 in the initiation/reinitiation of transcription. To determine if the observed decrease in TRO signal near the 5′ end of *CHA1* in the mutant was due to a failure to recruit RNAP II onto the promoter or due to a post-recruitment defect, we performed RNAP II density ChIP during the transcriptionally activated state of *INO1* and *CHA1* in *clp1-769-5* strain at permissive and non-permissive temperatures. RNAP II ChIP was performed using primer pairs A, B, C, D, E, and F as indicated in Figure 4A and 4D. Our results show that there was indeed a decrease in the density of RNAP II at the promoter region of both *INO1* and *CHA1* at elevated temperature (Figure 4B, lanes 1, 2 and Figure 4C, regions A, B; Figure 4E, lane 1 and Figure 4F, region A). There was no such decrease in the polymerase density at the promoter region of genes in the wild type cells at 37°C (Figure S5, B and E lanes 1 and 2; Figure S5, C and F). The RNAP II-ChIP experiment revealed a nearly 2-fold decrease in the polymerase signal at the 5′ end of *CHA1* in the mutant at 37°C (Figure 4F, region A). In contrast, TRO assay showed an at least 5-fold decrease in the polymerase signal near the promoter region of *CHA1* under identical conditions (Figure 2E, region C). This discrepancy could be attributed to the presence of transcriptionally inactive paused polymerase near the 5′ end of *CHA1* that can be detected by ChIP assay, but not by TRO assay. The overall conclusion of both the TRO and RNAP II-density ChIP results is that there is clearly a decrease in the amount of polymerase at the 5′ end of a gene in the *clp1* mutant at elevated temperature. A plausible interpretation of these results is that a functional CF1A complex facilitates the recruitment of RNAP II onto the promoter during transcription.

**Figure 5:**
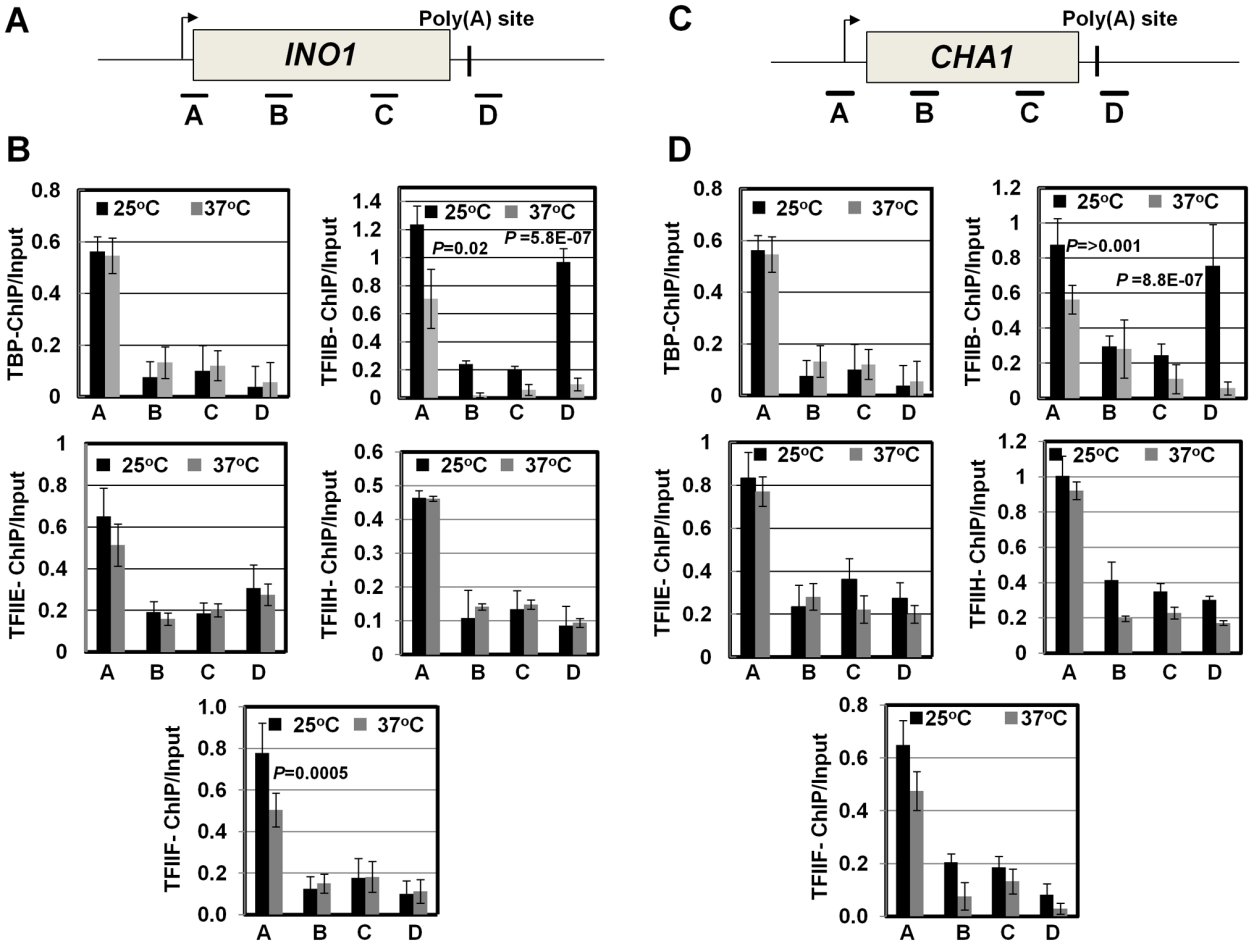
The promoter occupancy of the general transcription factors is not appreciably affected in the *clp1^ts^* mutant. (A, C) Schematic depictions of *INO1* and *CHA1* indicating the position of ChIP primer pairs. (B, D) Quantification of ChIP analysis showing crosslinking of the general transcription factors TFIID, TFIIB, TFIIF, TFIIE and TFIIH to different regions of *INO1* and *CHA1* in the *clp1* mutant at the permissive (25°C, black bars) and non-permissive (37°C, grey bars) temperatures. The results shown are an average of at least six independent PCRs from four separate immunoprecipitates from two independently grown cultures. Error bars indicate one unit of standard deviation.

Next we asked if CF1A-dependent recruitment of RNAP II on the promoter occurs during the initiation or reinitiation of transcription. During initiation of transcription, TFIID, TFIIB, TFIIA, TFIIF, RNAP II, TFIIE and TFIIH are recruited onto the promoter in that order to form the preinitiation complex (PIC) [56], [57]. The recruitment of RNAP II occurs subsequent to the formation of a TFIID-TFIIB-TFIIA complex on the promoter. This is followed by the binding of TFIIE and TFIIH to form the PIC. Following initiation of transcription, RNAP II along with TFIIF is released from the complex for elongation [58]. Simultaneously, TFIIB is also released from the complex, while the rest of the general transcription factors are left behind on the promoter forming a ‘scaffold’ that is used as a loading dock for the re-entry of RNAP II for reinitiation of transcription during subsequent transcription cycles. The composition of protein factors on the promoter, therefore, can distinguish an ‘initiation complex’ from the ‘reinitiation scaffold’ [14]. The initiation complex will contain all general transcription factors along with RNAP II, while the reinitiation scaffold will have general transcription factors with the exception of TFIIB and TFIIF and no RNAP II. Thus, to determine if CF1A-dependent recruitment of RNAP II was occurring during the initiation or reinitiation of transcription, we examined the promoter occupancy of *INO1* and *CHA1* for TFIID, TFIIB, TFIIF, TFIIE and TFIIH in *clp1-769-5* strain at the permissive and non-permissive temperatures by ChIP assay using primer pairs indicated in Figures 5A and 5C. Our results demonstrate that TFIID, TFIIB, TFIIF, TFIIE and TFIIH occupied the promoter region of both genes in the mutant at 25°C as well as 37°C (Figure 5B and 5D, region A black bar). Similar results were observed in the isogenic wild type strain (Figure S6). TFIIB also occupied the terminator region of both genes at 25°C (Figure 5B and 5D, region D grey bar for TFIIB-ChIP panel). The presence of TFIIB at the 3′ end of genes is linked to CF1A-dependent gene looping [18]. A decrease in TFIIB signal near the 3′ end of both *INO1* and *CHA1* was observed in the *clp1* mutant at 37°C (Figure 5B and 5D, region D grey bar for TFIIB-ChIP panel). This is in accord with the observed decrease in the TFIIB occupancy of the terminator region of transcriptionally active genes in the mutants of CF1A subunits [18]. A 25% decrease in the crosslinking of TFIIB and TFIIF to the promoter region of both *INO1* and *CHA1* was also observed in the mutant following the temperature shift to 37°C (Figure 5B and 5D, region A grey bar). This is in agreement with the reported release of TFIIB and TFIIF from the promoter following initiation of transcription [58]. There was no appreciable change in the promoter occupancy of the rest of the general transcription factors following a shift to elevated temperature, despite a decrease in the promoter-bound RNAP II signal. These results suggest that it is the reinitiation of transcription that is adversely affected in the *clp1-769-5* cells at elevated temperature. The overall conclusion of these results is that a functional CF1A complex is required for the recruitment of polymerase to the promoter for reinitiation of transcription. The possibility of CF1A complex being required for the recruitment of TFIIB and TFIIF for reinitiation cannot be ruled out.

### CF1A complex limits divergent anti-sense transcription at the promoter

During the transcription cycle, RNAP II in the promoter-bound initiation complex transcribes in the sense direction, producing mRNA. Genome wide analysis of transcribing polymerases has identified RNAP II molecules in the region just upstream of the transcription start site in most eukaryotic genes [45]–[48]. These upstream polymerases are involved in divergent anti-sense transcription, producing non-coding RNA (ncRNA). These promoter-initiated, anti-sense ncRNAs are capped, non-adenylated, heterogeneous in size and often belong to a class of RNA called CUTs (cryptic unstable transcripts) that are rapidly degraded by the RNA surveillance mechanism of the cell [59], [60]. Having already implicated CF1A complex in the sense-transcription of mRNA, we next asked if CF1A complex has a role in the regulation of divergent, anti-sense transcription of ncRNA. To address the issue, we performed strand-specific RT-PCR for *CHA1* in wild type and *clp1-769-5* mutant as described in [61]. In wild type cells, we could not detect promoter-initiated anti-sense transcripts under any condition (Figure 3C, region W). In the clp1-769-5 mutant also, no appreciable divergent anti-sense RNA could be detected at 25°C (Figure 3B, region W, black bar). At the elevated temperature, however, a 5-fold increase in the signal for promoter-associated anti-sense transcripts was observed in the mutant strain (Figure 3B, region W, grey bar). These results were corroborated by TRO assay, which detected the presence of transcriptionally engaged polymerase in the region upstream of *CHA1* in the mutant strain at 37°C (Figure 2C, lane 31; Figure 2E region A).

The increase in the level of divergent anti-sense transcripts initiating from the 5′ end of the gene in the mutant could be attributed either to the stabilization of the transcripts or to the synthesis of promoter-initiated anti-sense transcripts in the mutant. Since TRO assay detected the presence of transcriptionally active RNAP II just upstream of the promoter of *CHA1* in the *clp1* mutant at elevated temperature, it is reasonable to conclude that the observed anti-sense transcripts were not the consequence of stabilization of RNA, but the result of divergent anti-sense transcription initiating from the 5′ end of the gene. These results raise the possibility of a role for the CF1A complex in limiting the transcription of promoter-associated anti-sense ncRNA, thereby favoring transcription of mRNA in the sense direction. We therefore propose that the CF1A complex may have an additional role in providing directionality to otherwise bidirectional yeast promoters. Our results are in agreement with a recent report that showed an increase in promoter-initiated divergent anti-sense transcription in termination-defective mutants [49].

Thus, in the absence of a functional CF1A complex in the *clp1-769-5* mutant, the promoter-associated downstream transcription of mRNA in the sense direction as well as the divergent upstream transcription of anti-sense RNA, exhibited an aberrant pattern.

### A role for CF1A-dependent gene looping in promoter-associated transcription

**Figure 6:**
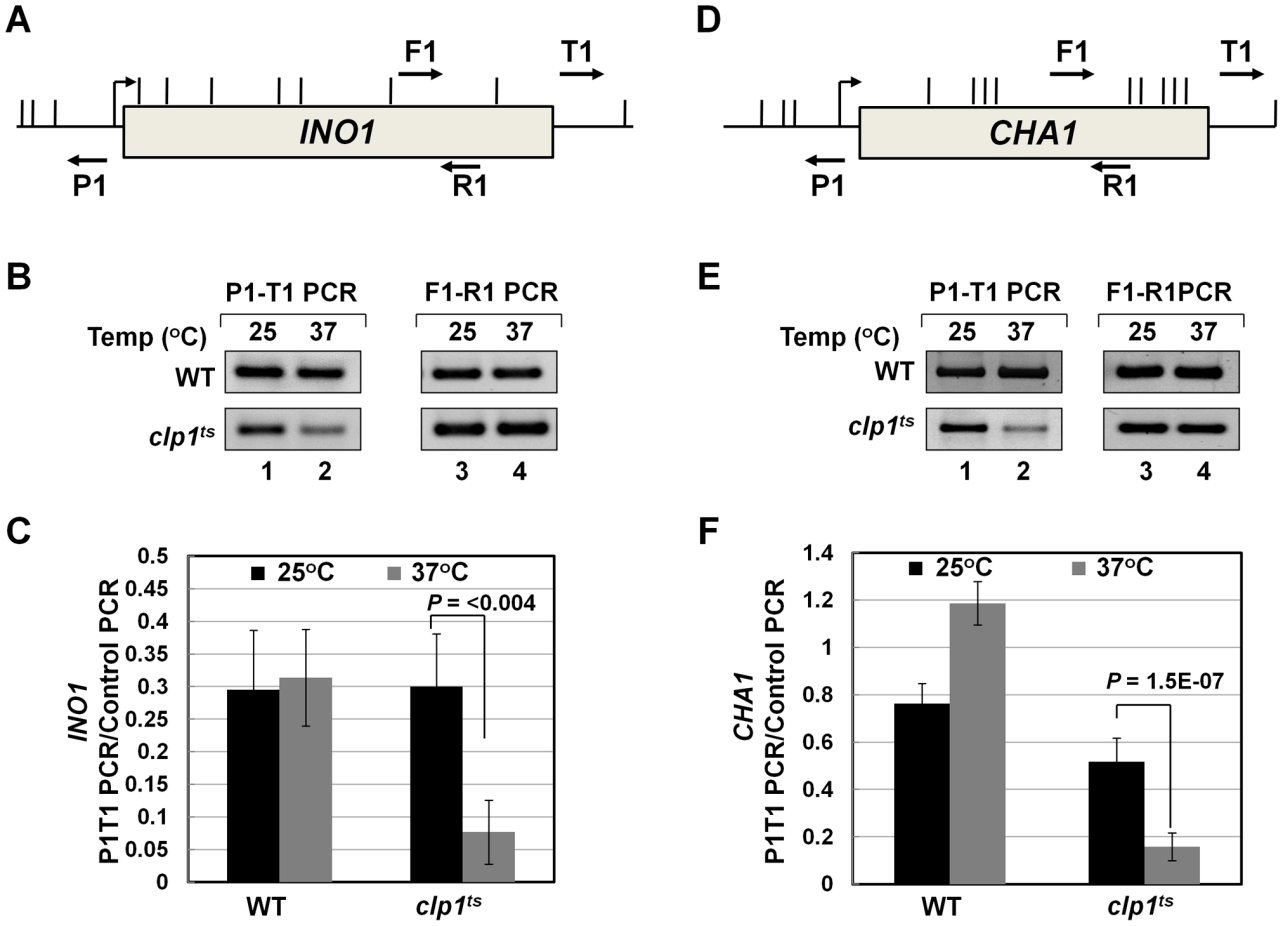
Gene looping of *INO1* and *CHA1* is compromised in *clp1^ts^* mutant. (A) and (D) Schematic depictions of *INO1* and *CHA1* indicating the position of restriction sites (vertical lines) and PCR primers (arrows) used in 3C analysis. (B) and (E) 3C analysis of *INO1* and *CHA1* to detect gene looping in the *clp1^ts^* mutant and the isogenic wild type strains at either permissive (25°C) or nonpermissive (37°C) temperatures. P1-T1 PCR reflects the looping signal while F1-R1 PCR represents the loading control indicating that an equal amount of template DNA was present in each of the 3C reactions. (C) and (F) Quantification of the 3C results shown in B and E. The results shown are an average of at least eight independent PCRs from four separate 3C replicates from two independently grown cultures. Error bars indicate one unit of standard deviation. WT = Wild type.

A logical interpretation of the results described above is that the CF1A complex is not merely contacting the 5′ end of transcriptionally active genes, but is also influencing early events in the transcription cycle. Next we asked how the CF1A complex is recruited to the 5′ end of a gene. The binding of CF1A complex to the 5′ end could be independent of its recruitment at the 3′ end of a gene. Alternatively, gene looping, which is the transcription-dependent interaction of the promoter and the terminator regions of a gene, may facilitate positioning of the terminator-bound CF1A complex at the 5′ end of a gene [30]. We have earlier demonstrated the role of CF1A subunits Rna14, Rna15 and Pcf11 in gene looping [18]. To corroborate the role of CF1A complex in gene loop formation, we performed 3C analysis of *INO1* and *CHA1* in the *clp1-769-5* mutant at the permissive and non-permissive temperatures. Gene looping was monitored by the P1-T1 primer pair shown in Figure 6A and 6D, by the method described in [62]. A distinct P1-T1 PCR signal was obtained for both *INO1* and *CHA1* when the mutant cells were grown at 25°C (Figure 6B and 6E, lane 1; Figure 6C and 6F, black bar). The P1-T1 looping signal decreased by about 4–6 fold following transfer of cells to 37°C (Figure 6B and 6E, lane 2; Figure 6C and 6F, grey bar). These results confirmed that a functional CF1A complex is indispensable for gene loop formation in budding yeast.

## Discussion

The CF1A complex, which is known to localize and operate at the 3′ end of RNAP II-transcribed genes in yeast, also contacts the 5′ end of genes. The promoter occupancy of the CF1A complex coincides with the gene assuming a looped conformation. We recently purified a holo-TFIIB complex that contained all the CF1 subunits and the general transcription factor TFIIB [18]. We showed that the holo-TFIIB complex mediates gene loop formation by simultaneously contacting the distal ends of a gene. Accordingly, gene looping was not observed in mutants of the Rna14, Rna15 and Pcf11 subunits of CF1 complex. Here we show that gene looping is abolished in the *clp1* mutant as well. Whether the presence of CF1A at the 5′ end is the cause or the effect of gene looping is still unclear, but it is quite evident that the CF1A subunits at the 5′ end of a gene affect early events during the transcription cycle. The CF1A-dependent gene loop juxtaposes the terminator region of a gene with its cognate promoter. This arrangement may facilitate binding of the RNAP II released from the terminator at the end of a transcription cycle to the promoter for starting the next round of transcription. Accordingly, we observed a 2-fold decrease in the RNAP II density at the promoter in the absence of a functional CF1A complex. Since the promoter occupancy of the general transcription factors, with the exception of TFIIB and TFIIF, remained unaltered in the *clp1* mutant, we propose that the CF1A complex, by virtue of its role in gene looping, affects reinitiation rather than initiation of transcription. The possibility of CF1A subunits playing a role in the initiation, however, still cannot be ruled out. A similar study carried out in a mammalian system found termination factors affecting initiation rather than reinitiation of transcription [42]. The mechanism of termination-dependent initiation, however, was not clear in that study. Here we propose that the CF1A-dependent gene looping may account for the termination-reinitiation link.

Since a majority of eukaryotic promoters are intrinsically bidirectional, there should be some mechanism in the cell to favor transcription of mRNA in the sense direction, over the anti-sense transcription of ncRNA [48]. We found that CF1A complex, while facilitating reinitiation in the sense direction, has an additional function in restricting transcription of the promoter-associated anti-sense RNA. The divergent, anti-sense transcription of ncRNA is widely believed to be terminated by the Nrd1-dependent pathway in yeast [63]. The CF1A complex, in general, is associated with the termination of mRNA synthesis by the poly(A)-dependent pathway [8], [9]. Our results suggest that CF1A complex may be involved in the termination of anti-sense ncRNA synthesis as well. These results are in agreement with a recent report that demonstrated crosslinking of mammalian termination factors Xrn2 and TTF2 to the 5′ end of genes and their involvement in limiting promoter-initiated anti-sense transcription [64]. The regulation of transcriptional directionality by Ssu72, which is a subunit of the CPF 3′ end processing complex in yeast, further corroborates our results [49]. The limiting of promoter-initiated anti-sense transcription may direct the polymerase to move in the sense direction, thereby producing mRNA. Thus, CF1A complex may be involved in providing directionality to bivalent promoters.

**Figure 7:**
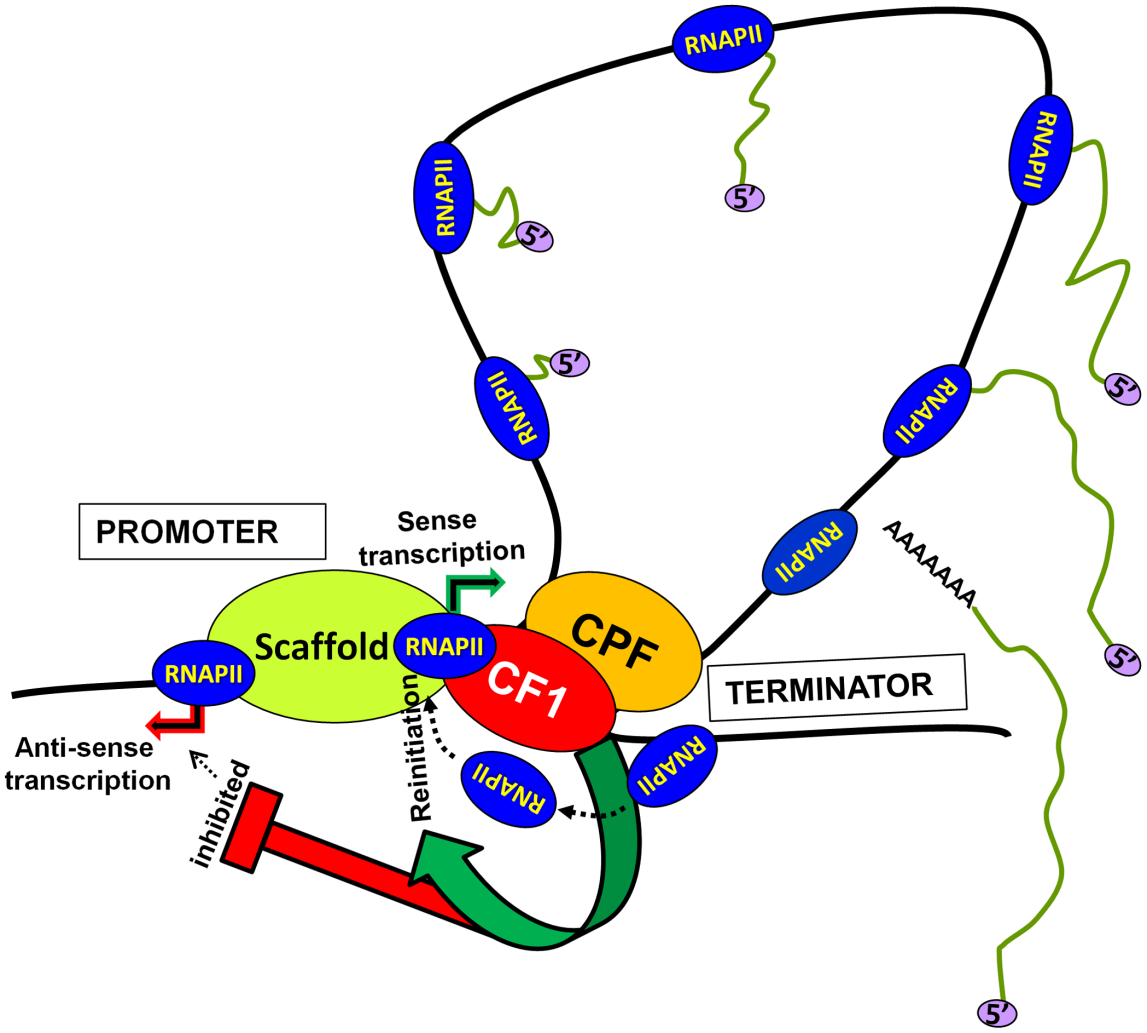
A model showing the role of CF1-dependent gene looping in promoting reinitiation of sense transcription and in limiting promoter-initiated divergent anti-sense transcription.

Based on these results we propose a model of transcription by RNAP II (Figure 7). The transcription-dependent promoter-terminator interaction places CF1A complex in the vicinity of the promoter. The promoter-bound CF1A affects transcription at two levels. First, CF1A-dependent termination releases RNAP II molecules from the 3′ end of gene near the promoter, thereby facilitating the recruitment of RNAP II to the promoter for reinitiation. Secondly, it provides directionality to the bidirectional promoter, thereby promoting the synthesis of mRNA over anti-sense ncRNA. Whether the CF1A complex limits promoter-initiated anti-sense transcripts by virtue of its termination activity needs further investigation. The net result is an upregulation of mRNA synthesis in the presence of a functional CF1A complex. Although a role for gene looping in facilitating transfer of polymerase from the terminator to the promoter for reinitiation has previously been hypothesized, this is the first instance where gene looping has actually been shown to help reinitiation of transcription.

## Materials and Methods

### Yeast strains

Yeast strains used in this study are listed in supplemental Table S1. SAM53, which contained the Myc-tag at the carboxy-terminal of Clp1 in BY4733 strain background, was constructed by transforming the parental strain with the PCR product amplified from pFA6-13Myc-TRP1. The temperature-sensitive mutant *clp1-769-5* was kindly provided by Dr. Philip Hieter. Strains NAH20, NAH21, NAH22, NAH31, NAH32 and NAH33 were derived from the temperature sensitive *clp1-769-5* strain by adding either the Myc or the Tap-tag at the carboxy terminus of an initiation factor or a termination factor. Strains NAH20 (Myc-tagged TFIIB), NAH21 (Myc-tagged Rna14), NAH22 (Myc-tagged Pcf11) and NAH25 (Myc-tagged Rna15), which contained the Myc-tag at the carboxy-terminus of the indicated factor, were constructed by transforming the *clp1-769-5* strain with the PCR product amplified from pFA6-13Myc-KanMX6. For TAP-tagging of the general transcription factors, first the temperature-sensitive *clp1-769-5* strain was made *trp1^−^* by replacing *TRP1* with a *KanMX* cassette that was PCR amplified from pUG6. Next a TAP-tag was inserted at the carboxy-terminus of TFIIH subunit Ccl1 (NAH31), TFIIF subunit Tfg2 (NAH33) and TFIIE subunit Tfa2 (NAH32) by transforming the *clp1-769-5-(trp1)* strain with the TAP-cassette amplified from plasmid pBS1479.

### Cell culture

Cultures were started by inoculating 5 ml of YP-dextrose medium with colonies from a freshly streaked plate, and grown at 25°C overnight with constant shaking. Next morning, overnight grown cultures were diluted (1∶100 dilution for the temperature-sensitive strains, and 0.5∶100 dilution for the wild type strains) to an appropriate volume and grown to OD_600_∼0.4. The dilution was done in the appropriate synthetic complete-drop out medium. Induction was done for 2 hrs at 25°C before shifting the cells to 37°C for another 2 hours for the deactivation step. Usually, this takes the cells to OD_600_ of about 0.7–0.8. At this stage, the cells are ready for processing for RT-PCR, 3C, ChIP, or TRO assays.

### Transcription Run-On assay (TRO)

Transcription run-on (TRO) assay was performed by the modification of protocols described in Birse et al., 1997 and Hirayoshi and Lis, 1999 [65], [66]. For *CHA1*, WT and *clp1-769-5* cells were grown in 100 ml of synthetic complete medium containing ammonium sulfate until A_600_ reached 0.4. Cells were centrifuged and resuspended in 100 ml of synthetic media containing serine and threonine (1 g/l each) and induced for 2 hours at 25°C. 50 ml of the cultures were centrifuged and resuspended in 50 ml of pre-warmed (37°C) serine and threonine containing medium and deactivation was done at 37°C for 120 minutes. The cell pellet obtained from 50 ml of liquid culture was washed with 10 ml cold TMN buffer (10 mM Tris-HCl pH 7.5, 5 mM MgCl_2_, 100 mM NaCl) and resuspended in 940 µl of DEPC (Diethylpyrocarbonate)-treated cold water. To the cell suspension, 60 µl of 10% sarkosyl was added and incubation performed on ice for 25 min to permeabilize the cells. Permeabilized cells were recovered by a low-speed centrifugation (1.2×g, 6 minutes) and directly used in the run on transcription assay. Elongation of transcripts initiated *in vivo* was resumed by resuspending cells in 120 µl of 2.5× reaction buffer (50 mM Tris-HCl pH 7.5, 500 mM KCl, 80 mM MgCl_2_, 5 mM DTT), 45 µl of NTPs/RNase inhibitor mix (10 mM each of CTP, ATP, and GTP and 300 units of RNase Inhibitor), and 7 µl of [α-^32^P]-UTP (3000 Ci/mmol, 10 µCi/µl). The reaction mix was incubated at 30°C for 2 minutes to allow transcript elongation. The reaction was stopped by adding 1 ml of cold TMN buffer and quickly spun at low speed. The recovered pellet was resuspended in 350 µl of Trizol. About 250 µl of acid-washed glass beads were added and the cells were lysed by vigorous shaking for 5 minutes on an agitator at room temperature. After lysis, tubes were spun for 5 minutes at 13800×g. To the recovered supernatant, 700 µl of Trizol and 200 µl of Chloroform were added and the samples were vigorously shaken on a vortexer, left on the bench for 5 minutes, and centrifuged at high speed for 10 minutes.

To isolate RNA, the supernatant was extracted twice with phenol/chloroform (pH 4.2). Labeled RNA was precipitated by adding 0.1 volumes of 10 M LiCl, 0.1 volumes of yeast tRNA (80 mg/ml) and 2.5 volumes of 100% ethanol. The mix was incubated at −20°C for 20 minutes followed by centrifugation at maximum speed for 15 minutes. The RNA pellet was resuspended in 60 µl of DEPC-treated water and denatured by adding 5 µl of 2 M NaOH followed by incubation on ice for 5 minutes. The NaOH was then neutralized by adding 12 µl of sodium acetate/acetic acid mix (0.3 M sodium acetate pH 5.2 and 0.5 µl of glacial acetic acid) and boiling the contents for 5 minutes.

In parallel, DNA probes of about 200–300 bp each in length, spanning the desired regions of the *CHA1* gene, including the upstream and downstream regions, were obtained by PCR amplification (See Fig. 1A for the position of probes). 10 µg of probe was denatured by boiling in 0.1 N NaOH and 1 mM EDTA for 10 minutes to form single stranded DNA. The heat-denatured probes were then slot-blotted on a ZETA-probe GT membrane (*BIO-RAD*), according to manufacturer's instructions. Adsorbed DNA was crosslinked to the membrane by baking at 80°C in a vacuum oven for 30 minutes. The membrane was then prehybridized with 10 ml of hybridization solution (0.5M potassium phosphate pH 7.2, 7% SDS) at 55°C for at least 30 minutes. The denatured RNA in hybridization solution from the step described above was added to the prehybridized membrane. Labeled RNA was allowed to hybridize to the probe for 18–24 hours at 55°C in a rotator. After hybridization, the membrane was washed twice with 20 ml of a solution containing 0.1% SDS and 1XSSC for 7 minutes at 55°C, and twice with 20 ml of a solution containing 0.1% SDS and 0.1XSSC for 7 minutes at 55°C. After drying, the membrane was exposed to X-ray film overnight in an autoradiography cassette and the films were developed in a Kodak M35A X-OMAT system. All TRO signals were quantified using the GEL LOGIC 200 (KODAK) system and normalized with respect to the 18S control.

### Chromatin immunoprecipitation analysis (ChIP)

ChIP was performed as described in [16]. Primers used for ChIP-PCR are described in supplemental Table S2 and indicated in Figures 4A, 4D, 5A and 5C. RNAP II ChIP was performed using anti-Rpb3 antibodies obtained from Santa Cruz (Cat# sc-101614). For ChIP analysis of CF1 subunits Clp1, Rna14, Rna15 and Pcf11, a Myc-tag was inserted at the carboxy-terminus of each subunit, and ChIP was performed using anti-Myc antibodies obtained from Upstate Biotechnology (Cat# 06-549). ChIP of TFIID was performed using anti-TBP antibodies obtained from Santa Cruz (Cat# sc-33736). ChIP analysis of TFIIB was carried out using anti-Myc antibodies in a strain with a C-terminus Myc-tagged TFIIB. For ChIP of TFIIF, TFIIE and TFIIH, strains were constructed with a TAP-tag inserted at the carboxy-terminus of Tfg2, Tfa2, and Ccl1 subunits respectively, and ChIP was performed using IgG-Sepharose beads.

Crosslinking, cell lysis and isolation of chromatin was done as described in [16]. Chromatin preparation obtained above was sheared by sonication (15 pulses of 20 seconds each with 1 minute cooling after each pulse). Sonication was performed at the 25% duty cycle in a Branson digital sonifier. Following sonication, samples were centrifuged at 14,000 rpm for 15 minutes in a refrigerated microfuge. The pellet was discarded and the supernatant was used in subsequent steps. The amount of sonicated chromatin to be used for immunoprecipitation depended on the quality of the antibody and the amount protein (antigen) present in the chromatin preparation. Approximately 5–10 µg of appropriate antibody (the amount of antibody added need to be optimized for each antibody preparation) was added to the chromatin preparation and allowed to bind for 4 hours at 4°C with gentle shaking. The antigen-antibody complex was adsorbed onto 20 µl of Protein A-Sepharose beads (the beads should be pre-washed with FA-lysis buffer) for 1 hour with gentle shaking at 4°C.

The beads were washed successively with 1 ml each of FA-lysis buffer (two times), FA-lysis buffer containing 500 mM NaCl (two times), ChIP wash buffer (10 mM Tris-HCl pH 8.0, 250 mM LiCl_2_, 0.5% tergitol, 0.5% sodium deoxycholate and 1 mM EDTA) and TE buffer. All the washing steps were performed at room temperature. The beads were resuspended in 250 µl of ChIP elution buffer (50 mM Tris-HCl of pH 8.0, 10 mM EDTA and 1% SDS); incubated at 65°C for 20 minutes; briefly spun; and the supernatant was collected and incubated with 10 µg of DNase-free RNase for 15 minutes at 37°C. 20 µg proteinase K and 2.5 µl 10% SDS were added and the crosslinks were reversed by overnight incubation at 65°C. Samples were extracted with phenol-chloroform at least two times followed by ethanol precipitation of DNA using glycogen as a carrier. The DNA pellet was resuspended in 50 µl TE and used as template for PCR. Chromatin immunoprecipitated DNA was PCR amplified (30 cycles) by appropriate primer pairs, and subjected to quantification and statistical analysis as described below. Each experiment was repeated with at least two independently grown cultures.

### 3C analysis

3C experiments were performed exactly as described previously [62]. The primers used for 3C analysis are shown in supplemental Table S2. A 50 ml cell culture was grown as described above. Cells were formaldehyde crosslinked for 15 minutes at 25°C. The crosslinked crude chromatin was digested with restriction endonuclease(s) (Alu1 for *INO1*; NlaIV and Alu1 for *CHA1*). After restriction digestion, the reaction volume was diluted by 7.5 fold to minimize intermolecular ligation in the next step. Ligation reactions were performed at room temperature for 90 minutes. The crosslinks were reversed by incubating at 65°C overnight. DNA was extracted with phenol-chloroform followed by ethanol precipitation. 300 ng of DNA was used as template in the PCR using the P1-T1 divergent primer pair as indicated in Figures 6A and 6D. Control PCR products were generated using a convergent primer pair (F2-R1). PCR and detection of products were performed exactly as described in [62]. Each experiment was performed with at least four independently grown cultures. The P1-T1 PCR signals are normalized with respect to F2-R1 PCR signals.

### Transcription analysis

Isolation of total RNA and transcription analysis was performed by RT-PCR using oligo-dT primer at the reverse transcription step as described previously (El Kaderi et al 2009). The RT-PCR primers are shown in supplemental Table S2. A minus-RT control (without reverse transcriptase) was always performed to ensure that the RT-PCR signal was not coming from contaminating DNA. The RT-PCR results were normalized with respect to the 18S rRNA control that is transcribed by RNAP I and requires a different set of transcription factors.

### Strand-specific RT-PCR

Strand-specific RT-PCR was performed to distinguish between sense and anti-sense transcripts. Total RNA for this procedure was extracted using Trizol reagent. The cell pellet was resuspended in 500 µl of Trizol. Acid-washed glass beads (about 250 µl) were added to the cell suspension. Cells were lysed by vigorous shaking for 10 minutes on an agitator at 4°C. Whole cell lysate was recovered by puncturing the bottom of the tube with a 22-guage needle, placing it on the top of a 15 ml pre-chilled centrifuge tube and centrifuging at 300×g for 2 minutes. The filtrate was transferred into a chilled 1.5 ml microfuge tube and 500 µl more Trizol reagent was added. After adding 200 µl of chloroform, tubes were vigorously agitated and left on the bench for 5 minutes. The tubes were then centrifuged at high speed for 10 minutes. The supernatant was extracted two times with an equal volume of phenol/chloroform (pH 4.3), followed by an extraction with chloroform only. RNA was precipitated using 0.1 volumes 10 M LiCl and 3 volumes cold ethanol in the presence of glycogen as a carrier. The precipitated RNA was collected by centrifugation at 14220×g on a table-top centrifuge for 15 minutes. The air-dried RNA pellet was resuspended in 50 µl of DEPC-treated water and the concentration was estimated using a spectrophotometer.

Strand specific RT-PCR was now performed as described in [16]. 1 µg of RNA was used to make cDNA using strand-specific primers for *CHA1* as shown in Figure 2A. Primers A_s_, B_s_, C_s_ and D_s_ were used to reverse-transcribe sense mRNA, while A_as_, B_as_ and C_as_ primers were used for reverse transcription of anti-sense transcripts. This was followed by PCR amplification of cDNA for regions W, X, Y and Z using primer pairs A_as_-B_s_, A_as_-A_s_, B_as_-C_s_ and C_as_-D_s_ respectively. A minus-RT control (without reverse transcriptase) was always performed to ensure that the strand-specific RT-PCR signal was not due to contaminating DNA in the RNA preparation. RT-PCR results were normalized with respect to the 18S rRNA control that is transcribed by RNAP I and requires a different set of transcription factors.

### Quantification

The quantification was performed as described in [62]. In ChIP, 3C and RT-PCR experiments described above, PCR products were fractionated on a 1.5% agarose gel and visualized by ethidium bromide using the Gel Logic 200 system. The net intensity of the bands was calculated using the Kodak 1D software. Using the scaled net intensities, a minimum of eight trials were analyzed under the Univariate ANOVA model in the SPSS statistical software to verify that there was no significant gel interaction (P<0.05). Each trial was also duplicated to ensure that there was no significant trial interaction (P<0.05). Scaled net intensities were then used to generate ratio data comparing the experimental test with that of the control PCR, which was then used to generate the mean and standard deviation as shown in the graphs. For all the quantification graphs, the error bars represent one unit of standard deviation based on at least eight independent PCRs from four separate IPs or 3C reactions or reverse-transcribed RNA samples from two independently grown cultures. For TRO, quantification was done with four independent replicates.

## Supporting Information

Figure S1 The amounts of the general transcription factor TFIIB and CF1A subunits Pcf11, Rna14, Rna15 are not altered in the *clp1^ts^* mutant at the non-permissive temperature. *clp1^ts^* mutant cells were grown at 25°C and then shifted to 37°C for 2 hours. Following cell lysis, Myc-tagged TFIIB or CF1A subunits were immunoprecipitated using anti-Myc antibodies from the cells grown at 25°C and 37°C. Equal amounts of protein were used in the immunoprecipitation. Immunoprecipitated proteins were fractionated on SDS-polyacrylamide gels and Western blot analysis was performed. (A) Western blot analysis for the cells grown at 25°C. (B) Western blot analysis for the cells grown at 37°C. Clp1 could not be detected in the cell lysate following the temperature shift to 37°C, while only a marginal change in the amounts of TFIIB and other CF1A subunits was observed in the mutant cells.
(TIF)

Figure S2 The recruitment of the CF1A subunits onto the *INO1* and *CHA1* genes is adversely affected in the *clp1^ts^* mutant at elevated temperature. (A, C) Schematic depictions of *INO1* and *CHA1* indicating the position of ChIP primer pairs. (B, D) ChIP analysis showing crosslinking of the CF1A subunits Rna14, Pcf11 and Rna15 to the 5′ and 3′ end of *INO1* and *CHA1* in the *clp1* mutant at 25°C and 37°C.
(TIF)

Figure S3 The recruitment of the CF1A subunits onto the *INO1* and *CHA1* genes remains unaffected in the wild type cells at elevated temperature. (A, C) Schematic depictions of *INO1* and *CHA1* indicating the position of ChIP primer pairs. (B, D) ChIP analysis showing crosslinking of the CF1A subunits Rna14, Pcf11 and Rna15 to the 5′ and 3′ end of *INO1* and *CHA1* in the *clp1* mutant at 25°C and 37°C.
(TIF)

Figure S4 Clp1 is recruited to the promoter and the terminator regions of transcriptionally active *INO1* and *CHA1*. (A) and (D) Schematic depictions of *INO1* and *CHA1* showing the positions of ChIP primer pairs. (B) and (E) ChIP analysis showing cross-linking of Clp1 to different regions of *INO1* and *CHA1* following 120 minutes of induction. (C) and (F) Quantification of the data shown in B and E respectively. Error bars indicate one unit of standard deviation.
(TIF)

Figure S5 RNAP II density in the promoter region remains unaffected in wild type cells at the elevated temperature. (A, D) Schematic depictions of *INO1* and *CHA1* showing the positions of ChIP primer pairs. (B, E) ChIP analysis showing polymerase density in different regions of *INO1* and *CHA1* in the wild type cells at the permissive (25°C, black bars) and non-permissive (37°C, grey bars) temperatures. (C and F) Quantification of data shown in B and E respectively. The input signals represent DNA prior to immunoprecipitation. The results shown are an average of at least eight independent PCRs from four separate immunoprecipitations from two independently grown cultures. Error bars indicate one unit of standard deviation. IP = immunoprecipitate.
(TIF)

Figure S6 The recruitment of the general transcription factors at the promoter of *INO1* and *CHA1* remains unaffected in the wild type cells at the elevated temperature. (A, C) Schematic depictions of *INO1* and *CHA1* indicating the position of ChIP primer pairs. (B, D) ChIP analysis showing crosslinking of the general transcription factors TFIID, TFIIB, TFIIF, TFIIE and TFIIH to different regions of *INO1* and *CHA1* in the wild type cells at 25°C and 37°C.
(TIF)

Table S1 List of strains used in this study.
(PDF)

Table S2 List of all the PCR primers used in this study.
(PDF)
