# Supplementary figures and images for: A Role for CF1A 3′ End Processing Complex in Promoter-Associated Transcription
Source: PLoS Genet. 2013 Aug 15;9(8):e1003722. doi: 10.1371/journal.pgen.1003722 (PMC3744418; doi:10.1371/journal.pgen.1003722)

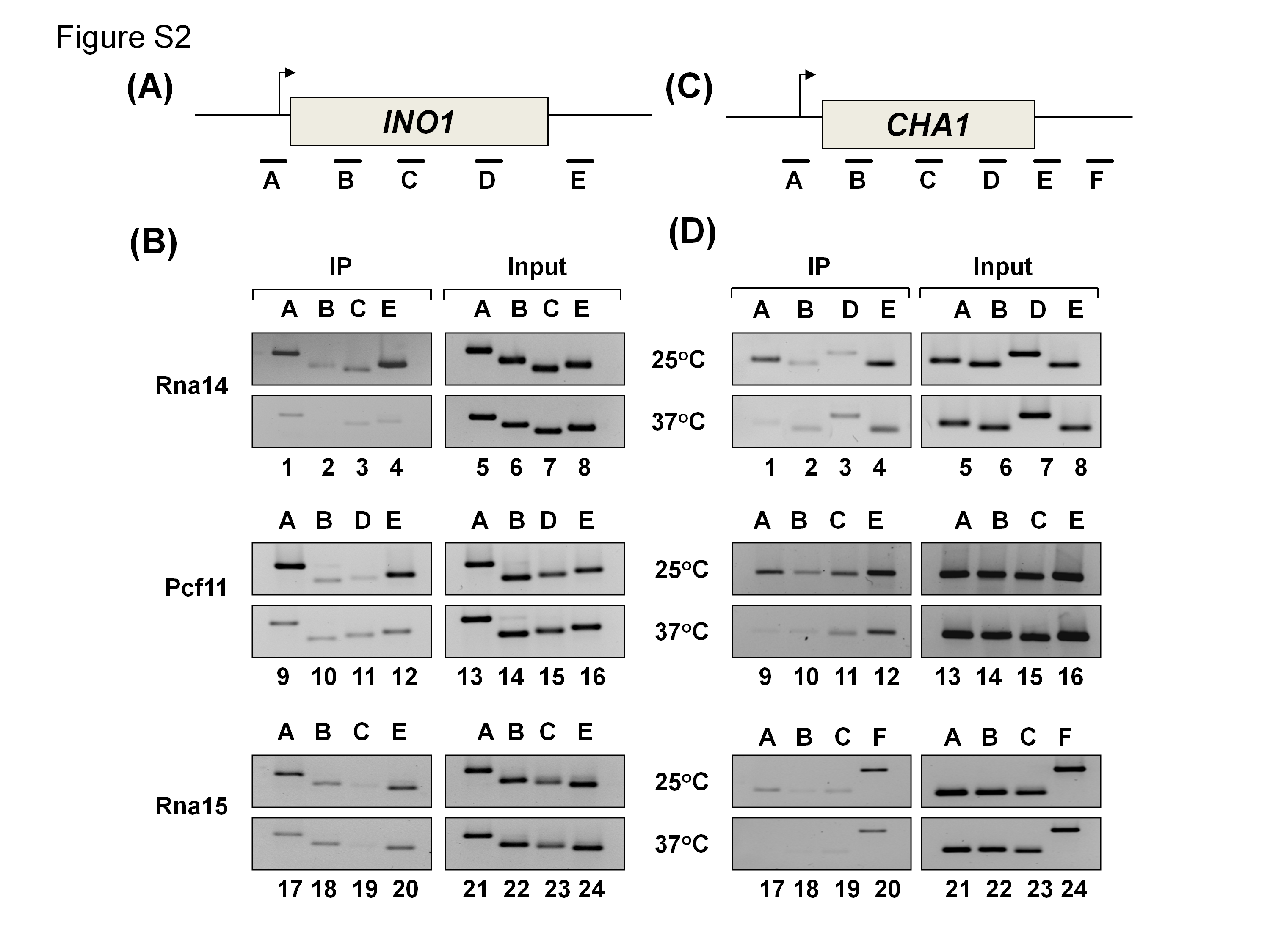

Supplement: Figure S2 — The recruitment of the CF1A subunits onto the INO1 and CHA1 genes is adversely affected in the clp1ts mutant at elevated temperature. (A, C) Schematic depictions of INO1 and CHA1 indicating the position of ChIP primer pairs. (B, D) ChIP analysis showing crosslinking of the CF1A subunits Rna14, Pcf11 and Rna15 to the 5′ and 3′ end of INO1 and CHA1 in the clp1 mutant at 25°C and 37°C. (TIF) [file pgen.1003722.s002.tif]

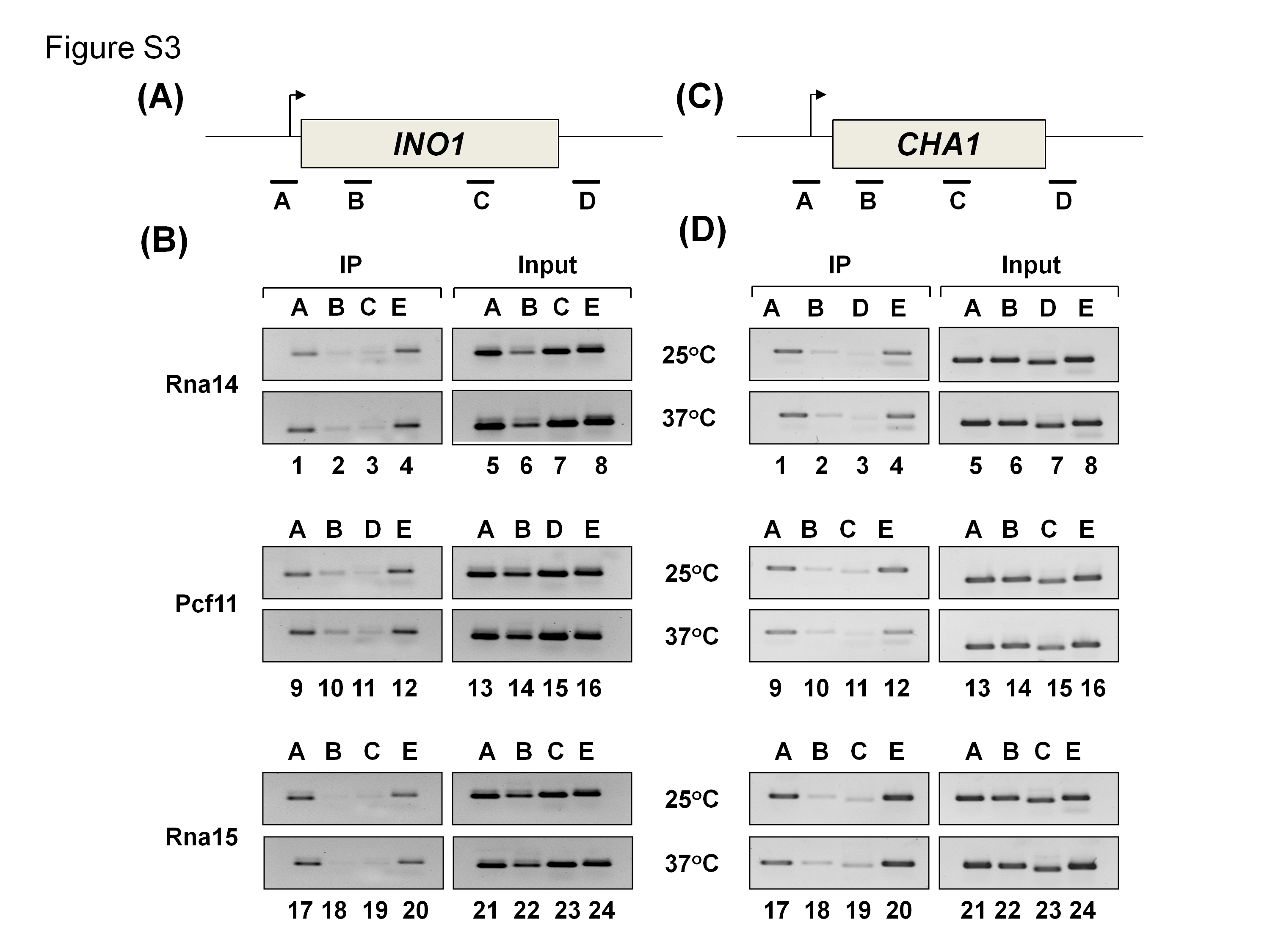

Supplement: Figure S3 — The recruitment of the CF1A subunits onto the INO1 and CHA1 genes remains unaffected in the wild type cells at elevated temperature. (A, C) Schematic depictions of INO1 and CHA1 indicating the position of ChIP primer pairs. (B, D) ChIP analysis showing crosslinking of the CF1A subunits Rna14, Pcf11 and Rna15 to the 5′ and 3′ end of INO1 and CHA1 in the clp1 mutant at 25°C and 37°C. (TIF) [file pgen.1003722.s003.tif]

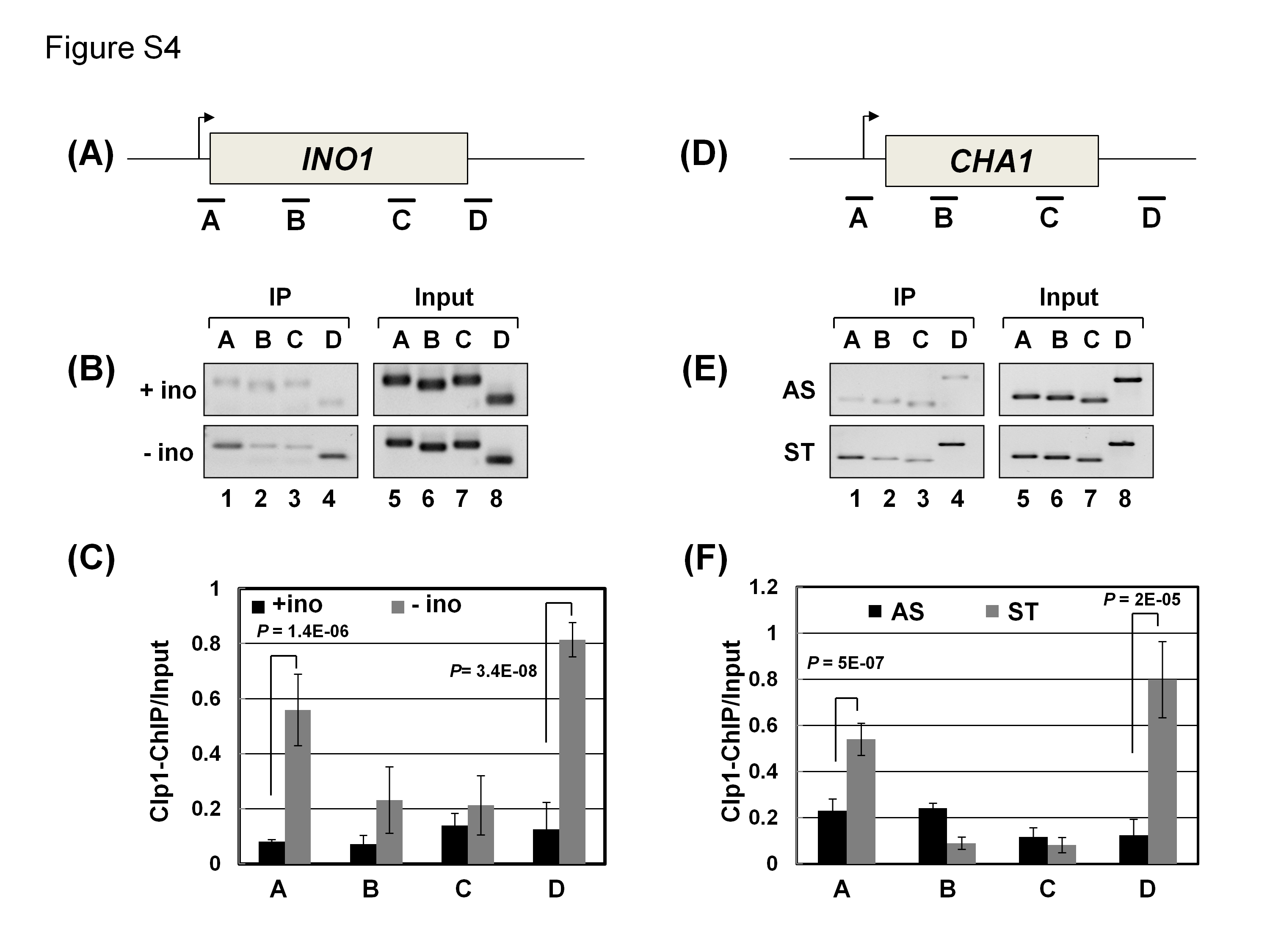

Supplement: Figure S4 — Clp1 is recruited to the promoter and the terminator regions of transcriptionally active INO1 and CHA1. (A) and (D) Schematic depictions of INO1 and CHA1 showing the positions of ChIP primer pairs. (B) and (E) ChIP analysis showing cross-linking of Clp1 to different regions of INO1 and CHA1 following 120 minutes of induction. (C) and (F) Quantification of the data shown in B and E respectively. Error bars indicate one unit of standard deviation. (TIF) [file pgen.1003722.s004.tif]

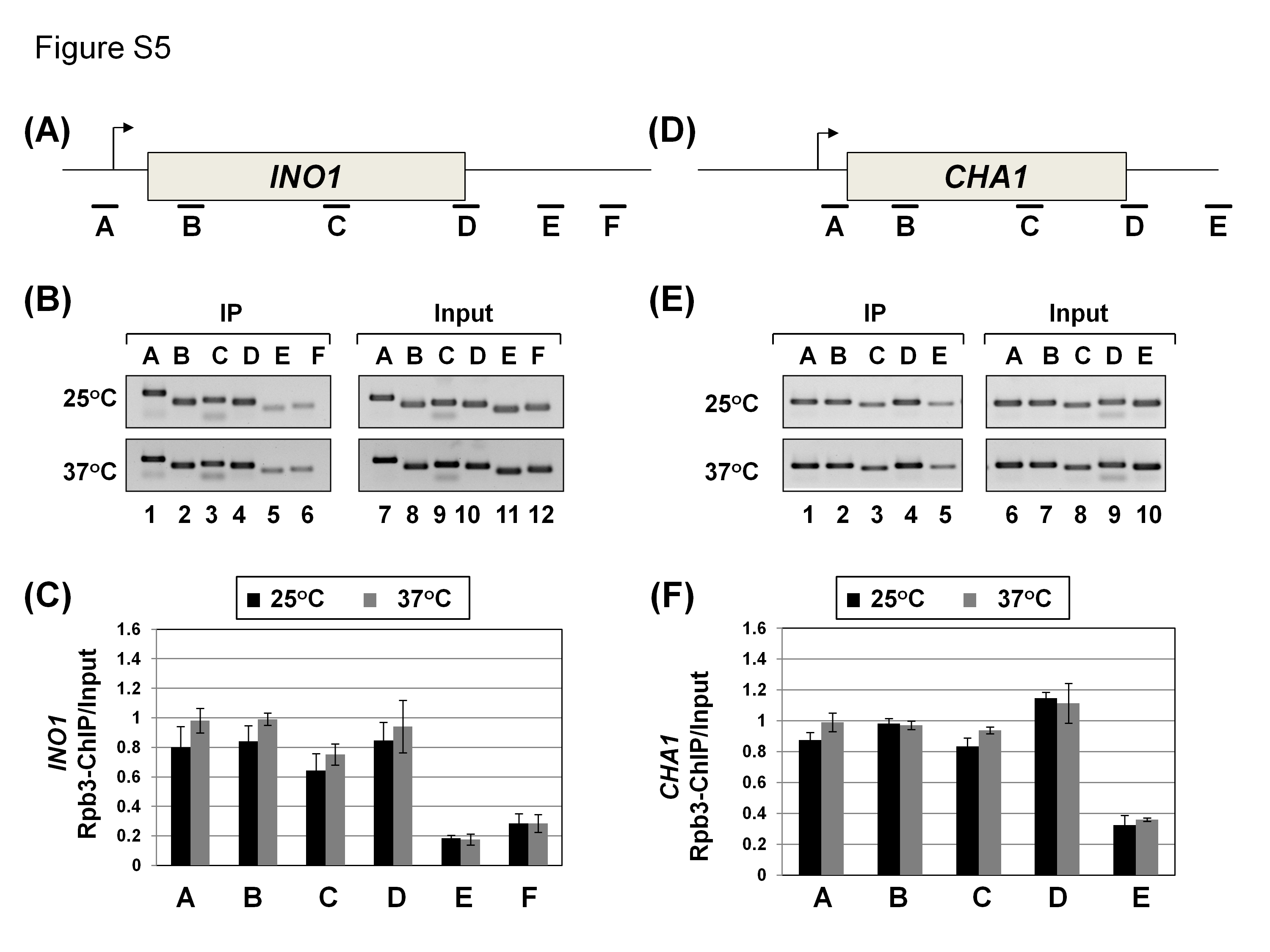

Supplement: Figure S5 — RNAP II density in the promoter region remains unaffected in wild type cells at the elevated temperature. (A, D) Schematic depictions of INO1 and CHA1 showing the positions of ChIP primer pairs. (B, E) ChIP analysis showing polymerase density in different regions of INO1 and CHA1 in the wild type cells at the permissive (25°C, black bars) and non-permissive (37°C, grey bars) temperatures. (C and F) Quantification of data shown in B and E respectively. The input signals represent DNA prior to immunoprecipitation. The results shown are an average of at least eight independent PCRs from four separate immunoprecipitations from two independently grown cultures. Error bars indicate one unit of standard deviation. IP = immunoprecipitate. (TIF) [file pgen.1003722.s005.tif]

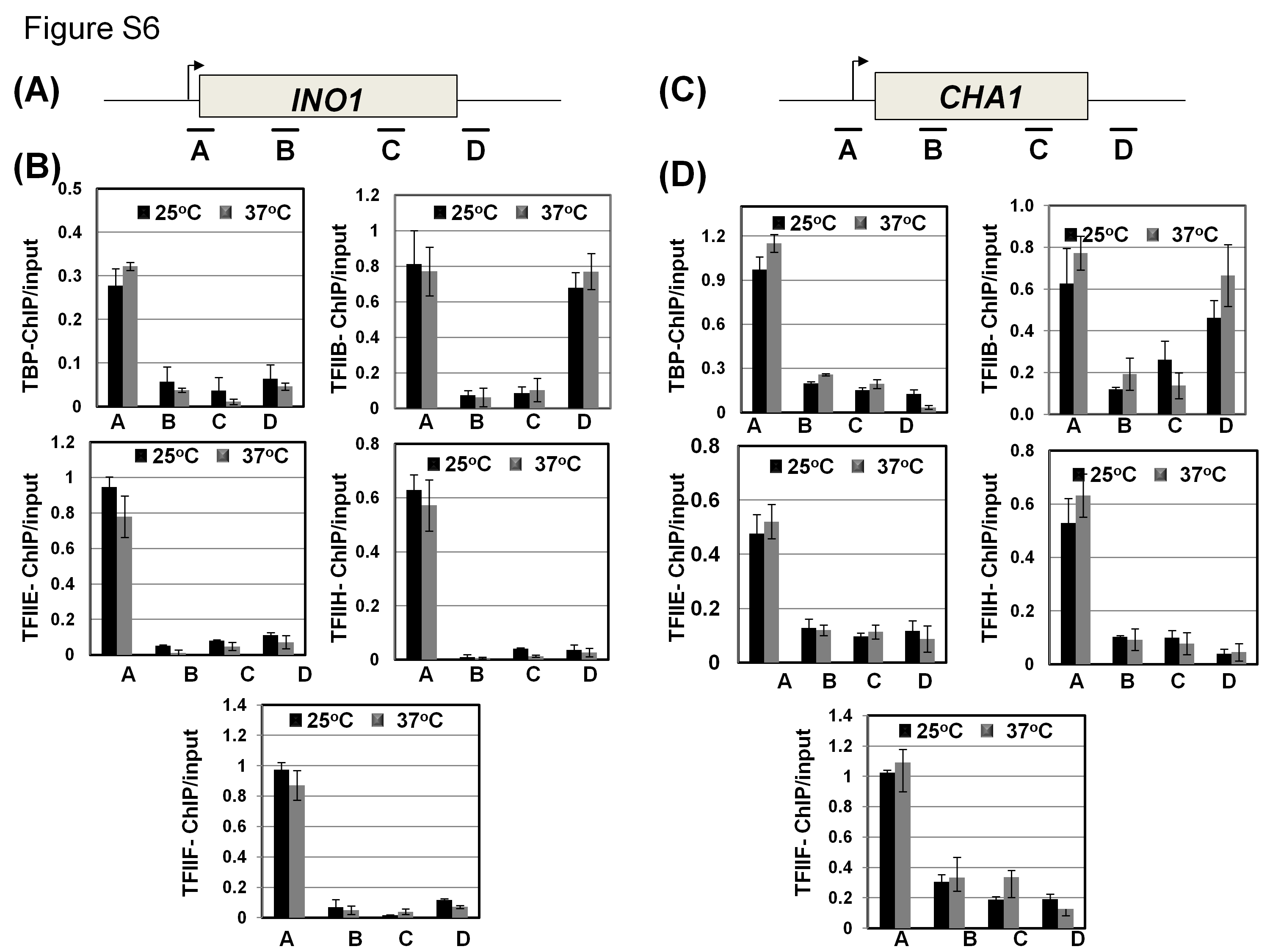

Supplement: Figure S6 — The recruitment of the general transcription factors at the promoter of INO1 and CHA1 remains unaffected in the wild type cells at the elevated temperature. (A, C) Schematic depictions of INO1 and CHA1 indicating the position of ChIP primer pairs. (B, D) ChIP analysis showing crosslinking of the general transcription factors TFIID, TFIIB, TFIIF, TFIIE and TFIIH to different regions of INO1 and CHA1 in the wild type cells at 25°C and 37°C. (TIF) [file pgen.1003722.s006.tif]
