# Supplementary material for: A Role for CF1A 3′ End Processing Complex in Promoter-Associated Transcription
Source: PLoS Genet. 2013 Aug 15;9(8):e1003722. doi: 10.1371/journal.pgen.1003722 (PMC3744418; doi:10.1371/journal.pgen.1003722)
Supplement: Table S1 — List of strains used in this study. (PDF) [file pgen.1003722.s007.pdf]

Table S1: STRAINS

Strain	***Genotype***	***Reference***
By4733	*MATa his3Δ200 trp1Δ63 leu2Δ0 met15Δ0 ura3Δ0*	
Clp1- 769-5	*MATa ura3Δ0 leu2Δ0 his3Δ1lys2Δ0can1Δ::LEU2-MFA1pr::His3 clp1ts::URA3*	[1]
SAM53	*BY4733, MATa his3Δ200 trp1Δ63 leu2Δ0 met15Δ0 ura3Δ0 CLP1-Myc (TRP)*	[2]
NAH20	*MATa ura3Δ0 leu2Δ0 his3Δ1 lys2Δ0can1Δ::LEU2-MFA1pr::His3 clp1-ts::URA3.SUA7(TFIIB)-Myc-KMX*	This study
NAH21	*MATa ura3Δ0 leu2Δ0 his3Δ1 lys2Δ0can1Δ::LEU2-MFA1pr::His3 clp1-ts::URA3 Rna14-Myc-KMX*	This study
NAH22	*MATa ura3Δ0 leu2Δ0 his3Δ1 lys2Δ0can1Δ::LEU2-MFA1pr::His3 clp1-ts::URA3 Pcf11-Myc-KMX*	This study
NAH25	*MATa ura3Δ0 leu2Δ0 his3Δ1 lys2Δ0can1Δ::LEU2-MFA1pr::His3 clp1-ts::URA3 Rna15-Myc-KMX*	This study
NAH26	*MATa ura3Δ0 leu2Δ0 his3Δ1lys2Δ0can1Δ::LEU2-MFA1pr::His3 clp1ts::URA3 Trp1Δ (KMX)*	This study
NAH31	*MATa ura3Δ0 leu2Δ0 his3Δ1 lys2Δ0can1Δ::LEU2-MFA1pr::His3 clp1-ts::URA3 CCL1-TAP (TRP)*	This study
NAH32	*MATa ura3Δ0 leu2Δ0 his3Δ1 lys2Δ0can1Δ::LEU2-MFA1pr::His3 clp1-ts::URA3 TFA2-TAP (TRP)*	This study
NAH33	*MATa ura3Δ0 leu2Δ0 his3Δ1 lys2Δ0can1Δ::LEU2-MFA1pr::His3 clp1-ts::URA3 TFG2-TAP (TRP)*	This study

REFERENCES

1. Ben-Aroya et al., (2008) Toward a comprehensive temperature-sensitive mutant repository of the essential genes of Saccharomyces cerevisiae. Mol Cell 25;30(2):248-58.

2. Medler et al., (2011) Evidence for a Complex of Transcription Factor IIB (TFIIB) with Poly(A) Polymerase and Cleavage Factor 1 Subunits Required for Gene Looping. JBC 286 ; 39 : 33709-33718
