# Supplementary material for: A Role for CF1A 3′ End Processing Complex in Promoter-Associated Transcription
Source: PLoS Genet. 2013 Aug 15;9(8):e1003722. doi: 10.1371/journal.pgen.1003722 (PMC3744418; doi:10.1371/journal.pgen.1003722)
Supplement: Table S2 — List of all the PCR primers used in this study. (PDF) [file pgen.1003722.s008.pdf]

Table S2: PRIMERS

CHA1 TRO-PRIMERS

Name	***Sequence***
CHA1 A	GATAGCCTCTTGCGACCTTATT
	CTTAACAGGAGCCGCCCAT
CHA1 B	GCCCCAGCGGAAATGTAA
	CATTCATATTTCAAGAAAAATTGTG
CHA1 C	GCGATGAGATAAGATAAAAGGGA
	GATTACCGATTCCTCTACTTTTGA
CHA1 D	AATTCAAAAGGACGGTAAAAGAT
	AAGGGATGAACATAAATGGGC
CHA1 E	GTTGGTGGAGGTGGTTTATACA
	TCTGGTGTTGTATTTGCGAGC
CHA1 F	GGTGGAAACGAATGGATGTC
	TCTTAGTGTTGTAACCCAAATGC
CHA1 G	GGAAGAAGCGTTGGATAGCAT
	CGTTTTGGATATGTTGATGCTTAC
CHA1 H	GCACAGAATTTGTATAAAGGGG
	GCTTTTCTTCACTTAGTAAGGATTAA
CHA1 I	GTTCCGTAATAATCTTCCCAGC
	CTGGGGTCTTCATTTGTGTCA

INO1 RNAP II-ChIP

Name	***Sequence***
INO1 A	GAAATATGCGGAGGCCAAG
	GGAGGTGATTGGAGCAATATTATC
NO1 B	GCTTGTTCTGTTGTCGGGTTC
	TCTTCGTAACTACAGCATTTTCG
NO1 C	GTATTAAACCGGTCTCCATTGC
	CCGACGGGCTTCATATATTTG
NO1 D	GATATCCAGAATTTCAAAGAAGAAAAC
	TATTCTGCGGTGAACCATTAATATAG
NO1 E	CTCATTTCAACGACTCTCTTTTTC
	ATGTTAAGTATATGTATTGATGGAAGG
NO1F	GGTAGATGCGAGAAAGTGCTG
	CTTCTTTCTCGTCCTCCTCCT
CHA1 A	GCCCCAGCGGAAATGTAA
	GATTACCGATTCCTCTACTTTTGA
CHA1 B	AATTCAAAAGGACGGTAAAAGAT
	AAGGGATGAACATAAATGGGC
CHA1 C	GGTGGAAACGAATGGATGTC
	TCTTAGTGTTGTAACCCAAATGC
CHA1 D	GGAAGAAGCGTTGGATAGCAT
	CCCCTTTATACAAATTCTGTGC
CHA1 E	GCACAGAATTTGTATAAAGGGG
	GCTTTTCTTCACTTAGTAAGGATTAA

CHA1- TFIIB –ChIP

Name	***Sequence***
CHA1 A	GATAGCCTCTTGCGACCTTATT
	CATTCATATTTCAAGAAAAATTGTG
CHA1 B	AATTCAAAAGGACGGTAAAAGAT
	AAGGGATGAACATAAATGGGC
CHA1 C	GGTGGAAACGAATGGATGTC
	TCTTAGTGTTGTAACCCAAATGC
CHA1 D	GGAAGAAGCGTTGGATAGCAT
	CCCCTTTATACAAATTCTGTGC

INO1 TFIIB-ChIP

Name	***Sequence***
INO1 A	GCTTGTTCTGTTGTCGGGTTC
	GGAGGTGATTGGAGCAATATTATC
INO1 B	GATATCCAGAATTTCAAAGAAGAAAAC
	TATTCTGCGGTGAACCATTAATATAG
INO1 C	GTATTAAACCGGTCTCCATTGC
	CCGACGGGCTTCATATATTTG
INO1 D	CTCATTTCAACGACTCTCTTTTTC
	GCACTTTCTCGCATCTACCTCA

CHA1-TBP –ChIP

Name	***Sequence***
CHA1 A	GCGATGAGATAAGATAAAAGGGA
	GATTACCGATTCCTCTACTTTTGA
CHA1 B	AATTCAAAAGGACGGTAAAAGAT
	AAGGGATGAACATAAATGGGC
CHA1 C	GGTGGAAACGAATGGATGTC
	TCTTAGTGTTGTAACCCAAATGC
CHA1 D	GGAAGAAGCGTTGGATAGCAT
	CGTTTTGGATATGTTGATGCTTAC

INO1 TBP-ChIP

Name	***Sequence***
INO1 A	GCTTGTTCTGTTGTCGGGTTC
	GGAGGTGATTGGAGCAATATTATC
INO1 B	GATATCCAGAATTTCAAAGAAGAAAAC
	TATTCTGCGGTGAACCATTAATATAG
INO1 C	GTATTAAACCGGTCTCCATTGC
	CCGACGGGCTTCATATATTTG
INO1 D	CTCATTTCAACGACTCTCTTTTTC
	GCACTTTCTCGCATCTACCTCA

CHA1-TFIIE-ChIP

Name	Sequence
CHA1 A	GCGATGAGATAAGATAAAAGGGA
	GATTACCGATTCCTCTACTTTTGA
CHA1 B	AATTCAAAAGGACGGTAAAAGAT
	AAGGGATGAACATAAATGGGC
CHA1 C	GGTGGAAACGAATGGATGTC
	TCTTAGTGTTGTAACCCAAATGC
CHA1 D	GGAAGAAGCGTTGGATAGCAT
	CCCCTTTATACAAATTCTGTGC

INO1 TFIIE-ChIP

Name	Sequence
INO1 A	GAAATATGCGGAGGCCAAG
	GGAGGTGATTGGAGCAATATTATC
INO1 B	GATATCCAGAATTTCAAAGAAGAAAAC
	TATTCTGCGGTGAACCATTAATATAG
INO1 C	GTATTAAACCGGTCTCCATTGC
	CCGACGGGCTTCATATATTTG
INO1 D	CTCATTTCAACGACTCTCTTTTTC
	GCACTTTCTCGCATCTACCTCA

CHA1- TFIIH-ChIP

Name	Sequence
CHA1 A	GCGATGAGATAAGATAAAAGGGA
	GATTACCGATTCCTCTACTTTTGA
CHA1 B	AATTCAAAAGGACGGTAAAAGAT
	AAGGGATGAACATAAATGGGC
CHA1 C	GGTGGAAACGAATGGATGTC
	TCTTAGTGTTGTAACCCAAATGC
CHA1 D	GGAAGAAGCGTTGGATAGCAT
	GGAAAAAATCAATACTAGCAAAATA

INO1 TFIIH-ChIP

Name	Sequence
INO1 A	GCTTGTTCTGTTGTCGGGTTC
	TCTTCGTAACTACAGCATTTTCG
INO1 B	TATTCTGCGGTGAACCATTAATATAG
	GTATTAAACCGGTCTCCATTGC
INO1 C	GTATTAAACCGGTCTCCATTGC
	CCGACGGGCTTCATATATTTG
INO1 D	GACAAAGAGGCAATAGTTCAAAAG
	CTCATTTCAACGACTCTCTTTTTC

CHA1-TFIIF-ChIP

Name	***Sequence***
CHA1 A	GCGATGAGATAAGATAAAAGGGA
	GATTACCGATTCCTCTACTTTTGA
CHA1 B	AATTCAAAAGGACGGTAAAAGAT
	AAGGGATGAACATAAATGGGC
CHA1 C	GGTGGAAACGAATGGATGTC
	TCTTAGTGTTGTAACCCAAATGC
CHA1 D	GGAAGAAGCGTTGGATAGCAT
	GGAAAAAATCAATACTAGCAAAATA

INO1-TFIIF-ChIP

Name	***Sequence***
INO1 A	GAAATATGCGGAGGCCAAG
	GGAGGTGATTGGAGCAATATTATC
INO1 B	GATATCCAGAATTTCAAAGAAGAAAAC
	TATTCTGCGGTGAACCATTAATATAG
INO1 C	GTATTAAACCGGTCTCCATTGC
	CCGACGGGCTTCATATATTTG
INO1 D	CTCATTTCAACGACTCTCTTTTTC
	GCACTTTCTCGCATCTACCTCA

CHA1-RT-PCR

Name	***Sequence***
CHA1 A	AATTCAAAAGGACGGTAAAAGAT
CHA1 B	AAGGGATGAACATAAATGGGC
18 S F	GGAATAATAGAATAGGACGTTTGG
18 S R	GTTAAGGTCTCGTTCGTTATCG

INO1-RT PCR

Name	***Sequence***
INO1 A	GATATCCAGAATTTCAAAGAAGAAAAC
INO1 B	TATTCTGCGGTGAACCATTAATATAG
18 S F	GGAATAATAGAATAGGACGTTTGG
18 S R	GTTAAGGTCTCGTTCGTTATCG

CHA1-CCC

Name	***Sequence***
*CHA1*P1	GATTACCGATTCCTCTACTTTTGA
*CHA1*T1	GTAAGCATCAACATATCCAAAACG
CHA1 F	AATTCAAAAGGACGGTAAAAGAT
CHA1 R	AAGGGATGAACATAAATGGGC

INO1-CCC

Name	***Sequence***
INO1 P1	GAACCCGACAACAGAACAAGC
INO1 T1	GTTGAGGTAGATGCGAGAAAGTG
INO1 F	GATATCCAGAATTTCAAAGAAGAAAAC
INO1 R	TATTCTGCGGTGAACCATTAATATAG

CHA1-Clp1p-Myc-ChIP

Name	Sequence
CHA1 A	GCGATGAGATAAGATAAAAGGGA
	GATTACCGATTCCTCTACTTTTGA
CHA1 B	AATTCAAAAGGACGGTAAAAGAT
	AAGGGATGAACATAAATGGGC
CHA1 C	GGTGGAAACGAATGGATGTC
	TCTTAGTGTTGTAACCCAAATGC
CHA1 D	GGAAGAAGCGTTGGATAGCAT
	CCCCTTTATACAAATTCTGTGC

INO1-Clp1p-Myc-ChIP

Name	Sequence
INO1 A	GAATATTGAACTTATTTAATTCACATGG
	GGAGGTGATTGGAGCAATATTATC
INO1 B	GTATTAAACCGGTCTCCATTGC
	CCGACGGGCTTCATATATTTG
INO1 C	GATATCCAGAATTTCAAAGAAGAAAAC
	TATTCTGCGGTGAACCATTAATATAG
INO1 D	CTCATTTCAACGACTCTCTTTTTC
	ATGTTAAGTATATGTATTGATGGAAGG

CHA1-Rna14-TAP-ChIP

Name	Sequence
CHA1 A	GCCCCAGCGGAAATGTAA
	CATTCATATTTCAAGAAAAATTGTG
CHA1 B	AATTCAAAAGGACGGTAAAAGAT
	AAGGGATGAACATAAATGGGC
CHA1 C	GTTGGTGGAGGTGGTTTATACA
	TCTTAGTGTTGTAACCCAAATGC
CHA1 D	GGAAGAAGCGTTGGATAGCAT
	CGTTTTGGATATGTTGATGCTTAC

INO1-Rna14-TAP-ChIP

Name	Sequence
INO1 A	GCTTGTTCTGTTGTCGGGTTC
	GGAGGTGATTGGAGCAATATTATC
INO1 B	GATATCCAGAATTTCAAAGAAGAAAAC
	TATTCTGCGGTGAACCATTAATATAG
INO1 C	GTATTAAACCGGTCTCCATTGC
	CCGACGGGCTTCATATATTTG
INO1 D	CTCATTTCAACGACTCTCTTTTTC
	GCACTTTCTCGCATCTACCTCA

CHA1-Rna15-TAP-ChIP

Name	***Sequence***
CHA1 A	GCGATGAGATAAGATAAAAGGGA
	GATTACCGATTCCTCTACTTTTGA
CHA1 B	AATTCAAAAGGACGGTAAAAGAT
	AAGGGATGAACATAAATGGGC
CHA1 C	GGTGGAAACGAATGGATGTC
	TCTTAGTGTTGTAACCCAAATGC
CHA1 D	GGAAGAAGCGTTGGATAGCAT
	CCCCTTTATACAAATTCTGTGC

INO1-Rna15-TAP-ChIP

Name	***Sequence***
INO1 A	GAAATATGCGGAGGCCAAG
	GGAGGTGATTGGAGCAATATTATC
INO1 B	GATATCCAGAATTTCAAAGAAGAAAAC
	TATTCTGCGGTGAACCATTAATATAG
INO1 C	GTATTAAACCGGTCTCCATTGC
	CCGACGGGCTTCATATATTTG
INO1 D	CTCATTTCAACGACTCTCTTTTTC
	GCACTTTCTCGCATCTACCTCA

CHA1-Pcf11-TAP-ChIP

Name	***Sequence***
CHA1 A	GCGATGAGATAAGATAAAAGGGA
	GATTACCGATTCCTCTACTTTTGA
CHA1 B	AATTCAAAAGGACGGTAAAAGAT
	AAGGGATGAACATAAATGGGC
CHA1 C	GGTGGAAACGAATGGATGTC
	TCTTAGTGTTGTAACCCAAATGC
CHA1 D	GGAAGAAGCGTTGGATAGCAT
	CGTTTTGGATATGTTGATGCTTAC

INO1-Pcf11-TAP-ChIP

Name	***Sequence***
CHA1 A	GCGATGAGATAAGATAAAAGGGA
	GATTACCGATTCCTCTACTTTTGA
CHA1 B	AATTCAAAAGGACGGTAAAAGAT
	AAGGGATGAACATAAATGGGC
CHA1 C	GGTGGAAACGAATGGATGTC
	TCTTAGTGTTGTAACCCAAATGC
CHA1 D	GGAAGAAGCGTTGGATAGCAT
	CGTTTTGGATATGTTGATGCTTAC

CHA1-Strand-Specific RT-PCR primers

Name	***Sequence***
A_{as}	CGAGTACTAATCACCGCGAAC
B_{as}	AATTCAAAAGGACGGTAAAAGAT
C_{as}	GGAAGAAGCGTTGGATAGCAT
A_s	AAGAGAAAACTGTATAAACATTTTCC
B_s	TCTCTTGTCTATCCAGCACTTAAAA
C_s	AAGGGATGAACATAAATGGGC
D_s	TGCTATCCAACGCTTCTTCC

Strain -making primers

Name	*Sequence*
5′ TFIIB-Myc-F2	TTGCTAATGGTGTAGTGTCTTTGGATAACTTACCGGGCGTTGAAAAGAA ACGGATCCCCGGGTTAATTAA
3'TFIIB-Myc-R1	CACGAGTACCCGTGCTTCTTGTTCCTATAATTTACTGTTTTATCACTTCA GAATTCGAGCTCGTTTAAAC
5′F1-RNA14-Myc-tag	TTTTAAATGATCAAGTAGAGATTCCAACAGTTGAGAGCACCAAGTCAGG TCGGATCCCCGGGTTAATTAA
3′R1-RNA14- Myc-tag	AGATGTGTTGGTATAAATATTCATATATACCTATTTATTAACGTAATGTTA GAATTCGAGCTCGTTTAAAC
5'F1- PCF11-Myc-tag	CTAATAGTGGCAAGGTCGGTTTGGATGACTTAAAGAAATTGGTCACAAA ACGGATCCCCGGGTTAATTAA
3′R1- PCF11-Myc-tag	TAATATAATATATAGTTATTAAATTTAAATGTATATATGCAGTTCTGCTC GAATTCGAGCTCGTTTAAAC
5' F2-RNA15-HA-tag	CTATTTGGGACTTAAAACAAAAAGCATTAAGGGGAGAATTTGGTGCATT TCGGATCCCCGGGTTAATTAA
3'RNA15-HA-tag	ATCATTGCGGAACCGCATTTTTTTTTTGTATTTTTGCCTCCCTAGTTTCA GAATTCGAGCTCGTTTAAAC
5′TFA2-TAP-C	TTACTAACACTCATATGACCGGTATCTTGAAAGATTATTCCCATAGAGTA TCCATGGAAAAGAGAAG
3'TFA2-TAP-C	CAGTCTCTTTAACCTAATATGCAAACGAAAATGATTTAATCAAAACAACC TACGACTCACTATAGGG
5′TFG2-TAP-C	GAGACGCGGAGGCTGACTTGGAAGATGAAATAGAAATGGAAGATGTCG TTTCCATGGAAAAGAGAAG
3'TFG2-TAP-C	CTCAAGAAACTGCGTAAATATAAAATTAATGAAGAAAATCTGATTGTCAA TACGACTCACTATAGGG
5′ CCL1-C-TAP	AGTTGAATGGAGAAGATACTTCGTCCACCGTTGAGAAAAAGCAAAAAAC ATCCATGGAAAAGAGAAG
3' CCL1-C-TAP	CTTAATCTATATATATATATATAAAACAGAAACCTACGGTAACAGAGCTGTT TACGACTCACTATAGGG
5′TRP1-KMX	TATTGAGCACGTGAGTATACGTGATTAAGCACACAAAGGCAGCTTGGA GTCAGCTGAAGCTTCGTACGC
3'TRP1-KMX	TGCAGGCAAGTGCACAAACAATACTTAAATAAATACTACTCAGTAATAAC GCATAGGCCACTAGTGGATCTG
